# Can supervised deep learning architecture outperform autoencoders in building propensity score models for matching?

**DOI:** 10.1186/s12874-024-02284-5

**Published:** 2024-08-02

**Authors:** Mohammad Ehsanul Karim

**Affiliations:** 1 School of Population and Public Health, University of British Columbia, 2206 East Mall, Vancouver, BC V6T 1Z3 Canada; 2 Centre for Advancing Health Outcomes, 588 - 1081 Burrard Street, Vancouver, BC V6Z 1Y6 Canada

**Keywords:** Machine learning, Propensity score, Deep learning, Causal inference, C18, 92D30, 62P10

## Abstract

**Purpose:**

Propensity score matching is vital in epidemiological studies using observational data, yet its estimates relies on correct model-specification. This study assesses supervised deep learning models and unsupervised autoencoders for propensity score estimation, comparing them with traditional methods for bias and variance accuracy in treatment effect estimations.

**Methods:**

Utilizing a plasmode simulation based on the Right Heart Catheterization dataset, under a variety of settings, we evaluated (1) a supervised deep learning architecture and (2) an unsupervised autoencoder, alongside two traditional methods: logistic regression and a spline-based method in estimating propensity scores for matching. Performance metrics included bias, standard errors, and coverage probability. The analysis was also extended to real-world data, with estimates compared to those obtained via a double robust approach.

**Results:**

The analysis revealed that supervised deep learning models outperformed unsupervised autoencoders in variance estimation while maintaining comparable levels of bias. These results were supported by analyses of real-world data, where the supervised model’s estimates closely matched those derived from conventional methods. Additionally, deep learning models performed well compared to traditional methods in settings where exposure was rare.

**Conclusion:**

Supervised deep learning models hold promise in refining propensity score estimations in epidemiological research, offering nuanced confounder adjustment, especially in complex datasets. We endorse integrating supervised deep learning into epidemiological research and share reproducible codes for widespread use and methodological transparency.

**Supplementary Information:**

The online version contains supplementary material available at 10.1186/s12874-024-02284-5.

## Background

**Challenges of estimating propensity scores based on parametric models**: The use of propensity score analyses in observational data analysis has been gaining popularity, thanks to its conceptual simplicity and the ease of diagnostic assessment [1]. However, inferring causality from propensity score analysis depends on several empirically unverifiable assumptions [2]. One critical assumption is the correct specification of both the propensity score and outcome models. In real-world applications, accurately determining the correct model specification for the propensity score analyses is extremely challenging for an analyst [3]. Notably, misspecifying the propensity score model can substantially bias the estimated treatment effect [4]. When estimating propensity scores using parametric models such as logistic regression, analysts must precisely understand and specify the correct functional form of the covariates, which might include quadratic, cubic terms, or interaction terms.

**Estimating propensity scores based on conventional machine learning models**: A variety of machine learning methods offer data-driven approaches to determine the specification of the model for estimating propensity scores, potentially reducing the bias in observational studies [5, 6]. Machine learning algorithms can automatically detect complex patterns and relationships, which traditional methods require extensive knowledge about the subject area. Methods such as shrinkage estimators (e.g., LASSO) help in reducing dimensions and stabilizing estimates [7, 8], but they may still rely on strong parametric assumptions. More flexible approaches, such as tree-based methods (e.g., gradient boosting machines), are effective in handling non-linear relationships and complex variable interactions [9, 10], though they may compromise the efficiency of treatment effect estimates [3]. Ensemble learning methods have been proposed, focusing on model averaging [11] or optimizing predictions from a diverse set of parametric and nonparametric learners [3].

**Estimating propensity scores based on deep learning models**: Deep learning models, free from the constraints of linearity and simplicity inherent in traditional statistical methods, present a promising avenue for estimating propensity scores, capable of capturing complex interactions and nonlinearities in observational data [12]. These models can process complex interactions and nonlinearities inherent in real-world data, crucial for accurate propensity score estimation in observational studies. The growing availability of computational resources has made using these intensive models more feasible, increasing their application in research. Their potential in enhancing the robustness and accuracy of causal inference in various fields is significant, though they pose challenges in interpretability and model complexity. Consequently, more researchers are exploring deep learning techniques in developing propensity scores where correct model-specification is an important issue [13–19]. However, the application of these methods in propensity score development is still limited [20], with evaluations often based on simplistic data generating mechanisms that do not fully exploit deep learning’s capabilities [14].

**Supervised versus unsupervised learning architectures within deep learning models**: Most machine learning or deep learning models used in the propensity score context were of supervised learning by nature. A recent work has proposed developing propensity score methods based on unsupervised learning algorithm, known as autoencoders [17]. An autoencoder, a neural network or deep learning variant of principal component analysis, not only reduces covariate dimensions but also accommodates complex covariate functional forms. Their simulations showed that autoencoder-based propensity scores yielded lower bias and mean squared error (MSE) in treatment effect estimation than multivariate regression, although the estimates suffered from significant under-coverage. This under-coverage, often resulting from discrepancies between empirical and model standard errors, indicates inaccuracies in variance estimation based on this suggested approach. The efficacy of autoencoder-based methods in low-dimension settings, typical in epidemiological studies, remains unclear.

**Aim**: This study aims to compare the performances of treatment effect estimators based on propensity score matching approaches, when the propensity scores will be built based on the following two deep learning algorithms: (1) a supervised deep learning learning algorithm that has a prediction focus and (2) an unsupervised deep learning learning (autoencoders) algorithm. For comparison purposes, we will also include (3) a parametric (based on logistic regression) and (4) a non-parametric (based on Multivariate Adaptive Regression Splines [MARS]) propensity score model, and compare their performance with the deep learning models. This comparison will be assessed via a plasmode simulation [21], inspired by the Right Heart Catheterization (RHC) dataset [22], incorporating a complex and realistic data generating mechanism.

To show the application of the approaches under consideration beyond simulation settings, we have also included analyses of the Right Heart Catheterization cohort, and provided the complete reproducible codes for the practitioners. For comparison purposes, we have also included results from Targeted Maximum Likelihood Estimation (TMLE) in this real-data application.

## Methods

### Data and simulation

**Right Heart Catheterization dataset**: We utilized the dataset from the ‘Study to Understand Prognoses and Preferences for Outcomes and Risks of Treatments’ (SUPPORT) [22]. Our focus was on evaluating the impact of RHC (binary exposure) on 1-month survival (death variable; binary outcome) within this dataset. The dataset comprises 5,735 subjects and 50 covariates previously used for adjustment in similar propensity score-based analyses [7]. We centered and scaled continuous variables within this dataset. Detailed descriptions of these covariates are available in Appendix §A.

**Plasmode simulation**: To rigorously evaluate the performance of the considered methods, we utilized a plasmode simulation framework, inspired by real-world data structures and complexities [21]. This framework, modeled after the empirical SUPPORT cohort study, uses resampling to sample from the observed covariates and exposure information (i.e., RHC) without modification. It effectively replicates key aspects of a real-world study, offering advantages over traditional Monte Carlo simulations. Our plasmode simulation included 1,000 iterations. For our base simulation scenario, we set the exposure (RHC) prevalence and event (death) rate at 30%, used a true odds ratio (OR) parameter of 0.7, and each simulation data had a sample size of 3,500. See Table 1 for the description of other scenarios under consideration.

**Table 1:** Simulation Scenarios for plasmode simulation based on the Right Heart Catheterization (RHC) study

Plasmode simulation scenario	Exposure prevalence	Outcome prevalence	True odds ratio	Sample size
(i) Frequent Exposure and Outcome (Base)	30%	30%	0.7	3,500
(ii) Rare Exposure and Frequent Outcome	5%	30%	0.7	3,500
(iii) Frequent Exposure and Rare Outcome	30%	5%	0.7	3,500
(iv) Large Sample Size for Base	30%	30%	0.7	5,000
(v) Null Effect for Base	30%	30%	1.0	3,500

**True data generating mechanism used in plasmode simulation**: The outcome variable (death) was modeled as a function of the exposure variable and a complex combination of covariates. This included main effects of all covariates, polynomial terms of the age variable up to the third order and the PaO2/FIO2 ratio up to the second order, a second-order interaction term between heart rate and mean blood pressure, and a third-order interaction among Glasgow Coma Score, Hematocrit, and Sodium. We also included the exponentiation of weight and the cosine of the APACHE score (see Appendix §B). A logistic regression model, reflecting this specified model, was used to generate the estimated outcomes in the simulation.

**Performance measures**: From this simulation, we derived several performance metrics: (1) bias, (2) average model standard error (SE; the average of estimated SEs obtained from a model over repeated samples), (3) empirical SE (the standard deviation of estimated treatment effects across repeated samples), (4) MSE, (5) coverage probability of 95% confidence intervals, (6) bias-eliminated coverage, and (7) Zip plot [23, 24].

### Estimators under consideration

Below, we describe four estimators based on propensity score matching, where model-specification differs by propensity score or exposure model estimation approaches. The software codes for executing each of the propensity score analyses strategies are described in details in Appendix §F.

**Fig. 1:**
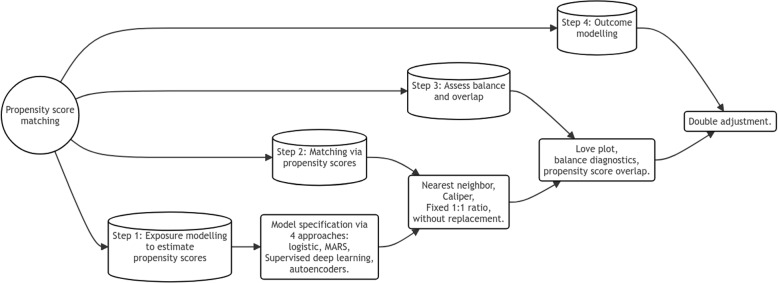
Steps followed for estimating treatment effect from the propensity score analyses

***Propensity Score Matching-based Estimators***:

We have illustrated the sequential process of propensity score matching analyses steps in Fig. 1. In our analysis, we employed four distinct approaches to estimate propensity scores (step 1): logistic regression, MARS, supervised deep learning, and autoencoders. During the estimation phase, all propensity score models were constructed using only the main effects of the 50 covariates, deliberately avoiding the complex model specification utilized in the plasmode data generating stage. This approach aims to reflect real-world data analysis scenarios more accurately.

After estimating the propensity scores, we applied the nearest-neighbor matching technique with respect to propensity scores without replacement, maintaining a 1:1 ratio between treated and untreated groups (step 2). We implemented a caliper set to 0.2 times the standard deviation of the logit-transformed estimated propensity scores [25]. For assessing balance of the matched cohort, we use standardized mean difference (SMD) (step 3, SMD of more than 0.25 was considered as an indication of imbalance in the data analysis [26]). Once the matched cohort was formed, we estimated the treatment effect using a logistic regression outcome model for death (step 4), doubly adjusted for all main effects of the covariates (same input variables that were used in the propensity score model development, not transformed in any way) to mitigate residual confounding [27, 28].

(i) **Propensity scores based on logistic regression**: The propensity scores were estimated using logistic regression, a widely employed method in this context [29]. This approach, in contrast to supervised deep learning or autoencoder-based models, does not incorporate validation steps.
(ii) **Propensity scores based on MARS**: MARS, a non-parametric regression technique, enhances linear models by adding the ability to capture nonlinear relationships and interactions [30]. It automatically determines optimal placement of knots and selects variables, including quadratic/cubic relationships and up to second-order interactions, for inclusion in the propensity score model. This selection is done using piecewise linear splines and a backward pruning method to prevent overfitting. While cross-validation is possible with MARS, it was not employed in our implementation [31]. The estimated propensity scores from this approach may not be always bounded between 0 and 1, and hence we applied truncation to make the estimated propensity scores between 0 and 1.
(iii) **Propensity scores based on Autoencoders**: We utilized a deep learning approach employing an autoencoder for feature extraction. It is particularly designed for dimensionality reduction and then feature extraction. It has a symmetric structure, and learns to compress the input data into a lower-dimensional representation (in the bottleneck layer) and then reconstructs the data from this representation (decoder part). See Appendix §C for the corresponding model architecture and compilation setup.
(iv) **Propensity scores based on supervised deep learning**: We employed a supervised deep learning framework, utilizing a sequential model. It has a more traditional architecture for classification tasks, and is primarily aimed at prediction (in this case, binary classification) based on the input features, with a focus on generalization and preventing overfitting. See Appendix §D for the corresponding model architecture and compilation setup.

**Fairness of the comparative analysis**: It is important to note that the supervised deep learning model included design features such as dropout, regularization, normalization, and kernel initialization, which were absent from the Autoencoder design. To address concerns regarding the fairness of our comparative analysis between these two approaches, we conducted additional experiments to evaluate the impact of these key design features. Specifically, we introduced two new versions: (a) a simplified version of the supervised deep learning model that omits dropout, regularization, normalization, and kernel initialization, and (b) an optimized version of the Autoencoder model that incorporates dropout layers with a rate of 0.3, L2 regularization with a penalty coefficient of 0.01, batch normalization layers, and the He normal initializer for kernel initialization, mirroring the supervised deep learning method.

***TMLE-based Estimators***:

Only for the real-world data analysis part, we have also used TMLE approach for comparison purposes [32, 33]. Once the propensity scores were estimated from each of the 4 above approaches under consideration (i.e., logistic regression, MARS, prediction focused supervised deep learning and autoencoders), we have used those in the TMLE framework instead of the propensity score matching. For the outcome model, to allow more flexibility, we have used a super learner for the outcome model estimation with 5 fold cross-validation, and used logistic regression and MARS as candidate learners [34]. We have obtained corresponding treatment effect estimates and associated $95\%$ confidence intervals from TMLE approach. Since TMLE is a double robust approach, variance estimation from this approach is known to be more suitable particularly when model-specification is hard to guess.

## Results

**Fig. 2:**
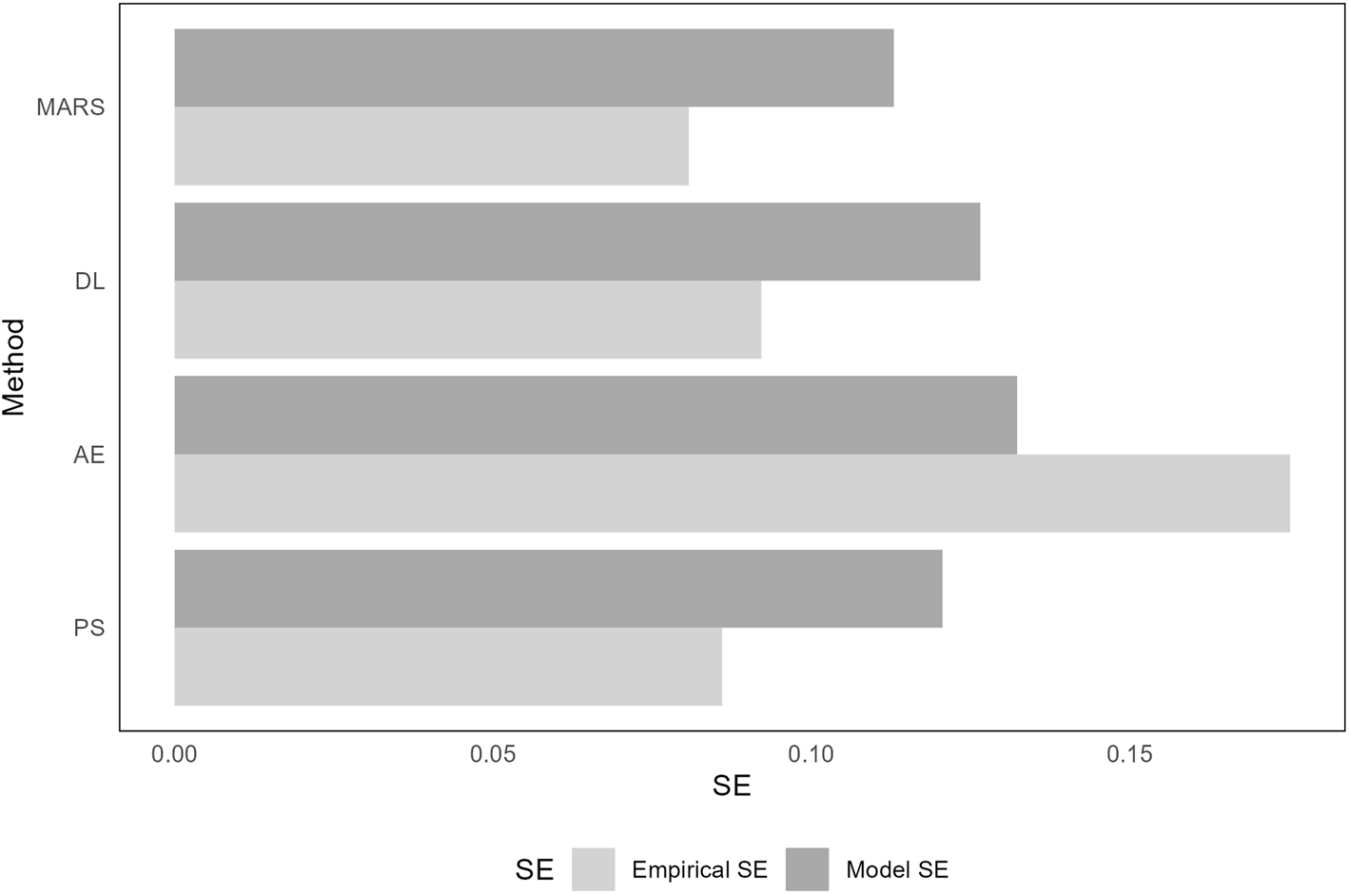
Comparing the empirical and average model standard errors from four propensity score estimation methods in the plasmode simulation based on the Right Heart Catheterization study in the presence of a frequent exposure (prevalence $30\%$) and a frequent outcome (prevalence $30\%$): Logistic Regression (PS), Autoencoders (AE), Deep Learning (DL), and Multivariate Adaptive Regression Splines (MARS). The results are derived from $1,000$ sets of plasmode simulation data, each with a sample size of $3,500$. The true target parameter was set to an odds ratio of $0.7$

**Base scenario**: The statistical properties of the estimated propensity scores, derived from four different methods evaluated in our base plasmode simulation studies, are presented in Table 2. Regarding bias, the performance of all methods was notably similar. Although the average model-based standard errors (SEs) were comparable across all approaches, a significant variation was observed in the empirical SEs. The empirical SE from the autoencoder approach was the highest, consequently leading to the greatest mean squared error (MSE). A comparison between the average model-based SEs and the empirical SEs from the same methods revealed that the empirical SE is substantially higher than the average model SE for the autoencoder approach. In contrast, for the other methods, the empirical SE is marginally lower than the average model SE (refer to Fig. 2). Consequently, in terms of coverage probability for the 95% confidence intervals, as well as their bias-eliminated counterparts, the autoencoder approach exhibited undercoverage, whereas the other approaches demonstrated overcoverage (see Appendix Figure E.1 [24]).

**Table 2:** Performance measures of the 4 different propensity score matching methods from the plasmode simulation based on the Right Heart Catheterization (RHC) study. The results (point Estimate of performance measures, and Monte Carlo SE) are derived from 1,000 sets of plasmode simulation data, each with a sample size of 3,500

Performance measure	PS	AE	DL	MARS
Bias	-0.0041 (0.0027)	0.0073 (0.0055)$\uparrow$	-0.0023 (0.0029)$\uparrow$	-0.0035 (0.0026)$\uparrow$
Empirical SE	0.0860 (0.0019)	0.1752 (0.0039)$\uparrow$	0.0921 (0.0021)$\uparrow$	0.0807 (0.0018)$\downarrow$
MSE	0.0074 (0.0003)	0.0307 (0.0018)$\uparrow$	0.0085 (0.0004)$\uparrow$	0.0065 (0.0003)$\downarrow$
Model-based SE	0.1206 (0.0001)	0.1323 (0.0002)$\uparrow$	0.1265 (0.0001)$\uparrow$	0.1130 (0.0001)$\downarrow$
Coverage	0.9940 (0.0024)	0.8900 (0.0099)$\downarrow$	0.9920 (0.0028)$\downarrow$	0.9940 (0.0024)
Bias-eliminated Coverage	0.9930 (0.0026)	0.8960 (0.0097)$\downarrow$	0.9920 (0.0028)$\downarrow$	0.9940 (0.0024)$\uparrow$

Here, *PS *propensity score based on logistic regression, *AE* propensity scores based on Autoencoders, *DL *propensity scores based on supervised deep learning, *MARS* Propensity scores based on Multivariate Adaptive Regression Splines, *SE* Standard Error, *MSE* Mean Squared Error, *Coverage *Coverage for nominal $95\%$ Confidence Interval. Arrows indicate the direction of the change in performance measures relative to the PS method: $\uparrow$ indicates an increase, and $\downarrow$ indicates a decrease

**Fig. 3:**
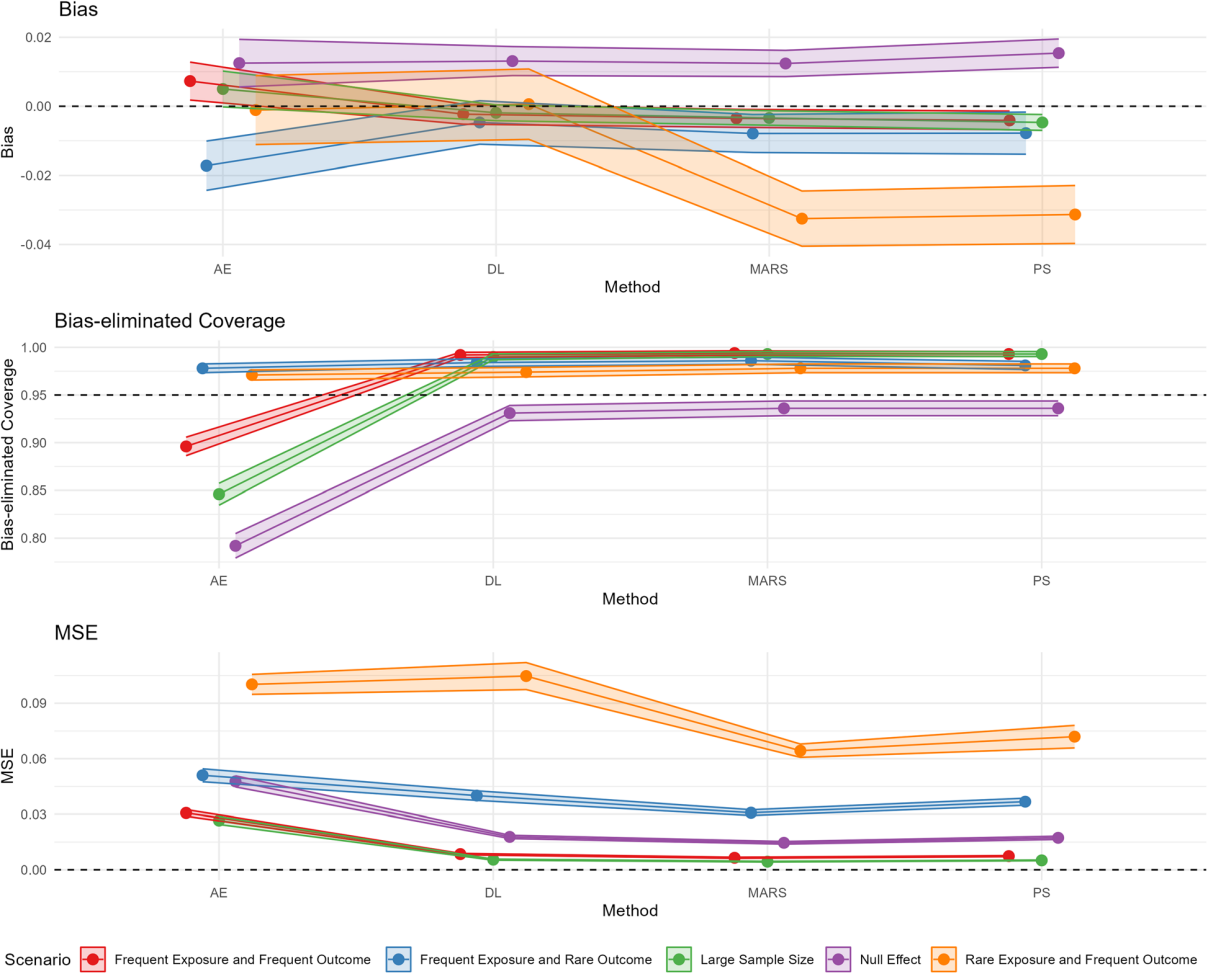
Plots comparing bias, bias-eliminated coverage probabilities and mean squared error [MSE] (point Estimates and Monte Carlo Standard Errors of corresponding performance measures) from four propensity score estimation methods in the plasmode simulation under different scenarios: Logistic Regression (PS), Autoencoders (AE), Deep Learning (DL), and Multivariate Adaptive Regression Splines (MARS). The results are derived from 1, 000 sets of plasmode simulation data

**Comparing all scenarios**: Results from the other simulation scenarios are shown in Fig. 3 and Appendix Figure E.2. The estimated bias exhibited similar patterns across different scenarios. As expected, propensity score-based methods performed poorly when exposure was rare [35]. However, when deep learning-based methods were used (both for supervised deep learning and autoencoders), the bias was notably low, even though the mean squared error estimates were very high for all methods under this scenario. Coverage and bias-eliminated coverage probabilities were notably poor for the base scenario, and the situation did not improve with a larger sample size or when the effect estimate was null. More detailed numerical results are presented in Appendix Tables E.1-E.5.

**Comparing other deep learning versions**: We have also presented extended results in Appendix Table E.1, where we included an additional version of the supervised deep learning model (naive) and another version of the autoencoders (optimized by adding the same design features, such as dropout, regularization, normalization, and kernel initialization). Our findings indicate that the inclusion of these design features somewhat improved the performance of the supervised deep learning model (DL) compared to its simplified counterpart (DL.n). Generally, autoencoder methods (with or without these added design features) perform poorly in terms of estimation of empirical standard error and coverage probabilities compared to either version of the supervised deep learning methods (see Appendix Table E.1). Particularly, the performance of the optimized autoencoder model (AE.o) was notably poorer compared to the original autoencoder model (AE) across several performance measures, including bias and MSE. Most notably, the optimized autoencoder model showed very poor results in terms of mean squared error, coverage, and bias-eliminated coverage. These results highlight that the inclusion of advanced design features did not uniformly benefit both models, underscoring the importance of model-specific tuning and evaluation.

## Real-world analysis

**Fig. 4:**
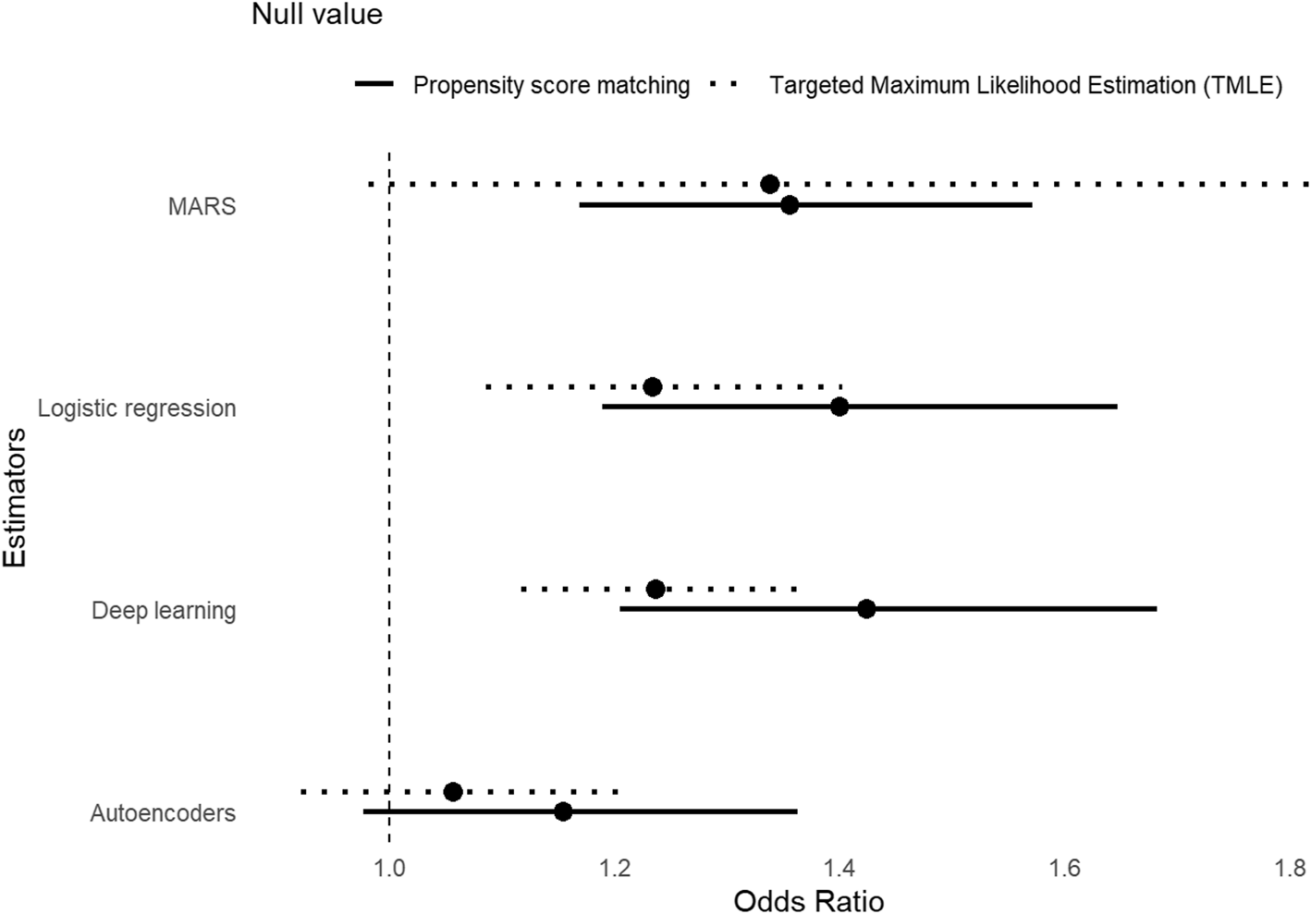
Analyses of the Study to Understand Prognoses and Preferences for Outcomes and Risks of Treatments study data to estimate the association between the Right Heart Catheterization and death based on propensity scores estimated from logistic regression (PS), Autoencoders (AE), supervised deep learning (DL), and Multivariate Adaptive Regression Splines (MARS). Estimates from Targeted Maximum Likelihood Estimation (TMLE) approach is also added corresponding to each approach for comparison purposes

**Estimates**: In analyzing the SUPPORT cohort data using propensity scores estimated from the four considered approaches, we calculated the treatment effects on the OR scale, along with their associated 95% confidence intervals. The results from the autoencoder approach did not yield significant results, whereas the other methods provided very similar OR estimates and confidence intervals (see Fig. 4). Therefore the conclusion from the autoencoder approach would be different compared to the other approaches. Reproducible software codes for this data analysis is presented in Appendix F, that includes diagnostic plots, such as love plot [36].

**Fig. 5:**
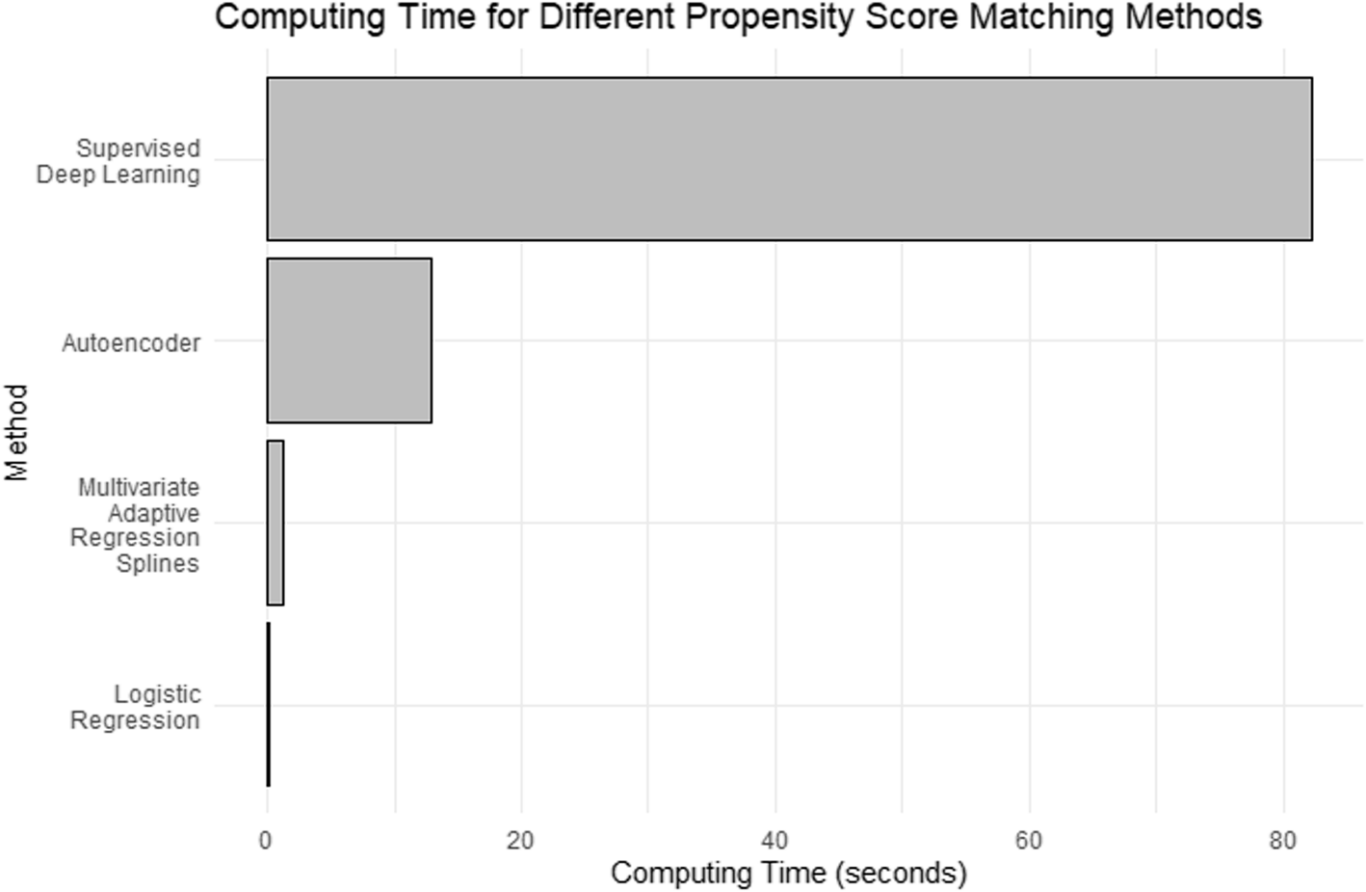
Computing Time for different propensity score matching methods: Logistic Regression, Multivariate Adaptive Regression Splines (MARS), Autoencoder, and Supervised Deep Learning, ordered by computing time

**Computing time**: Deep learning-based methods typically demand substantial resources and extended computing time. In our propensity score matching analysis, the total computing time required for each method was as follows: logistic regression took 0.18 seconds, MARS required 1.19 seconds, autoencoder necessitated 12.9 seconds, and the supervised deep learning approach took the longest at 82.3 seconds. These computing times are summarized in Fig. 5.

## Discussion

**Contextualizing the literature**: The performance of estimates from propensity score matching approaches often depends on the model specifications, including the selection of covariates and their functional form. This dependency can significantly influence the matching process and the resultant balance between the treated and control groups [37]. The iterative nature of propensity score matching also allows researchers considerable leeway in selecting matched samples, potentially introducing bias. Despite these concerns, propensity score matching (e.g., pair matching) continues to be a favored method for propensity score analysis in healthcare research [29, 38].

Deep learning methods, known for their proficiency in managing high-dimensional data, such as in medical image analysis [39], have shown considerable promise in epidemiological studies, even with tabular datasets [40]. The increasing application of deep learning methods across various healthcare research areas has recently sparked interest in their application to tabular health datasets. These methods are adept at automatically detecting various nonlinear and non-additive patterns. A recent study proposed a 1:1 propensity score matching approach (with nearest neighbor without replacement matching) based on an unsupervised deep learning approach: autoencoders [17]. It was found that autoencoder-based methods outperformed multivariate regression in terms of bias reduction. However, the study suggested that while autoencoders offer a data-adaptive method for propensity score computation, they may not provide substantial advantages over traditional machine learning methods in terms of confounding adjustment. In particular, it was noted that the autoencoder approach resulted in suboptimal coverage for the 95% confidence interval, achieving only 88.7%. Additionally, it was observed that increasing the number of layers in autoencoders tended to deteriorate the coverage probabilities.

In response to these findings, we assessed the performance of a prediction-focused supervised deep learning approach for propensity score modeling. The fundamental difference between this supervised and the previously proposed autoencoder lies in their core objectives: supervised deep learning model focuses on making accurate predictions, employing techniques to enhance model generalization, whereas autoencoder approach is more geared towards dimension reduction, and hence posing the risk of information loss during data condensation.

**Summary of the simulation findings**: Our plasmode simulation incorporated an outcome model with non-linearities, non-additivities, and complex functions such as exponentiation and cosine operations in the covariates. All methods, including a deep learning-based approach, showed comparable performance in terms of bias for the base scenario. However, variance estimates were slightly inaccurate for all methods, with the most significant issues arising in the autoencoder-based method, leading to under-coverage (89% in the base scenario), akin to previous findings [17]. In contrast, the supervised deep learning methods (with or without advanced design features) notably improved variance estimates and coverage probabilities.

Although our results indicate that supervised deep learning and conventional logistic regression exhibit similar performance in terms of bias in most scenarios we considered, deep learning methods have distinct advantages in specific scenarios. For instance, in settings with rare exposure, supervised deep learning and autoencoder methods demonstrated better performance compared to conventional methods (e.g., logistic regression and MARS). Additionally, supervised deep learning models are inherently capable of capturing complex, non-linear relationships and interactions among variables, which might not be easily modeled by logistic regression in more complex data settings. However, this does not mean that all deep learning methods have similar performance. For example, autoencoders performed worse than the supervised deep learning method in terms of coverage probability estimation in most settings. Furthermore, we observed the importance of model-specific tuning and evaluation when we added additional versions of deep learning models.

**Data analysis findings**: In actual data analysis, the ORs and 95% confidence intervals estimated by the autoencoder approach differed from those obtained with other propensity score matching-based methods. However, the estimates from the proposed supervised deep learning approach aligned more closely with those from the MARS and logistic regression methods. While the true data generating mechanism of the original dataset remains unknown, the similarity in results from logistic regression, MARS, and supervised deep learning approach may indicate a lack of complex associations among covariates.

In terms of variance estimation, double robust methods often offer advantages in the scenarios when specification of the model is hard to determine [6]. Since model-specification is the core issue, we also have included TMLE-based estimates in the comparison. To make the analyses results comparable, same propensity scores were used in both matching and TMLE-based approaches. The point estimates from the TMLE methods were always slightly lower than those from the matching methods. The influence-curve based $95\%$ confidence intervals from TMLE approaches were always associated with shorter confidence intervals compared to the confidence intervals from the matching methods, except for MARS approach. The MARS approach’s probability predictions are not always confined to the (0,1) interval, which could be a plausible reason for such deviation in the SE estimates.

**Future Direction**: While our study aimed to compare the performance of propensity score matching approaches when the propensity scores were generated from deep learning-based approaches (e.g., Autoencoders vs. supervised deep learning models), we acknowledge the potential value in exploring hybrid approaches. Specifically, one anonymous reviewer suggested that integrating the latent representations from the bottleneck layer of Autoencoders with supervised DL methods could offer an innovative avenue for enhancing propensity score estimation. This hybrid approach could potentially leverage the strengths of both unsupervised and supervised learning techniques, leading to improved performance. Future research could investigate this hybrid approach to further advance the field.

**Conclusion**: Our investigation reveals that a supervised deep learning approach for estimating propensity scores in observational studies demonstrates superior performance in variance estimation compared to unsupervised autoencoders, particularly in settings with complex data structures. As the field of causal inference continues to evolve, the integration of advanced machine learning techniques with traditional statistical methods holds the potential to significantly advance our understanding of treatment effects in observational data.

### Supplementary Information

Supplementary Material 1.

## Data Availability

The dataset utilized for this manuscript on right heart catheterization is publicly accessible and can be retrieved from https://hbiostat.org/data/. The corresponding software codes are available in the Appendix.

## Abbreviations

MSE: Mean Squared Error
SE: Standard Error
PS: Propensity Score
AE: Autoencoders
DL: Deep Learning
MARS: Multivariate Adaptive Regression Splines
SMD: Standardized Mean Difference
TMLE: Targeted Maximum Likelihood Estimation
RHC: Right Heart Catheterization
SUPPORT: Study to Understand Prognoses and Preferences for Outcomes and Risks of Treatments
