# Supplementary material for: Can supervised deep learning architecture outperform autoencoders in building propensity score models for matching?
Source: BMC Med Res Methodol. 2024 Aug 2;24:167. doi: 10.1186/s12874-024-02284-5 (PMC11295454; doi:10.1186/s12874-024-02284-5)

Supplementary Content

Can Supervised Deep Learning Architecture Outperform Autoencoders in building Propensity Score Models for Matching?

A Covariates in the Right Heart Catheterization study

A comprehensive set of 50 covariates was incorporated in our analyses to evaluate the Right Heart Catheterization (RHC) data. These covariates encompass a broad spectrum of demographic, clinical, and physiological variables. Appendix Table 1 in the Appendix E includes a summary of these covariates, stratified by RHC status.

1. Key demographic variables include age, sex, and race. Educational background and income levels, segmented into categories such as less than $11k, $25k to $50k, and over $50k, are also considered. Health insurance status is captured through several classifications, including Medicare, Medicare and Medicaid, private insurance, private insurance with Medicare, and those with no insurance.

2. Clinical or disease-specific variables are grouped under primary and secondary disease categories, encompassing a wide range of diagnoses such as respiratory, cardiovascular, neurological, gastrointestinal, renal, metabolic, hematologic, sepsis, trauma, and orthopedic conditions. Functional and health status indicators include the Duke Activity Status Index (DAS2D3PC), DNR status, cancer status (localized or metastatic), and the SUPPORT model estimate of 2-month survival probability. Additionally, the analysis accounts for a wide array of comorbidities, including acute myocardial infarction, peripheral vascular disease, various stages of cardiovascular symptoms, congestive heart failure, dementia, psychiatric conditions, chronic pulmonary disease, chronic renal disease, liver conditions, gastrointestinal bleeding, malignancies, immunosuppression, organ transplant, HIV positivity, diabetes, and connective tissue disease.

3. Physiological and laboratory measurements are extensively covered, including the Glasgow Coma Score, weight, temperature, mean blood pressure, respiratory rate, heart rate, PaO2/FIO2 ratio, PaCO2, pH level, white blood cell count, hematocrit, sodium, potassium, creatinine, bilirubin, and albumin levels. Hospital-related variables such as transfer status from another hospital and myocardial infarction history are also included.

B Outcome Model-specification in the Plasmode Simulation

The data generating model for the plasmode simulation includes

- main effects of all covariates stated above,
- polynomial terms of the age variable up to the third order (`age + age^2 + age^3`)
- the PaO2/FIO2 ratio up to the second order (`pafi1 + pafi1^2`),
- a second-order interaction term between heart rate and mean blood pressure (`hrt1 * meanbp1`), and
- a third-order interaction among Glasgow Coma Score, Hematocrit, and Sodium (`scoma1:hema1:sod1`),
- the exponentiation of weight (`exp(wtkilo1)`) and
- the cosine of the APACHE score (`cos(aps1)`).

C Model Architecture for the Autoencoders

The autoencoder was designed as a sequential model with a mirrored encoder-decoder architecture. The encoder consisted of two dense layers with 100 and 50 neurons, each using ReLU activation. The bottleneck layer, crucial for feature compression, had 30 neurons with ReLU activation. The decoder mirrored the encoder, expanding the compressed features back to the original dimension. The output layer used sigmoid activation with neurons equal to the number of input features.

The dataset was divided into training (80%) and testing (20%) subsets for model training and internal validation. The model was compiled using mean squared error as the loss function and the Adam optimizer with an initial learning rate. Key callbacks included early stopping to prevent overfitting, learning rate scheduling for adaptive learning rate adjustments, model checkpointing to save the best model, and logging of training metrics.

The autoencoder was trained over 150 epochs with a batch size of 32. The model's performance was evaluated on the test set, assessing reconstruction accuracy. Post-training, the learning rate was updated for potential subsequent training phases. Feature extraction was conducted by isolating the last layer's output, providing a compressed representation of the input data. These features were then utilized in propensity score model building.

Appendix Table C.1: Autoencoder Model Architecture

Component	Layer.Type	Units	Activation
Encoder Layer 1	Dense	100	ReLU
Encoder Layer 2	Dense	50	ReLU
Bottleneck Layer	Dense	30	ReLU
Decoder Layer 1	Dense	50	ReLU
Decoder Layer 2	Dense	100	ReLU
Output Layer	Dense	Input data dimension	Sigmoid

Appendix Table C.2: Compilation and Training Setup of the Autoencoder

Property	Value
Loss Function	Mean Squared Error
Optimizer	Adam
Learning Rate	0.0001 (Initial), Adjusted by Learning Rate Scheduler
Callbacks	Early Stopping, Learning Rate Scheduler, Model Checkpoint, CSV Logger
Epochs	150
Batch Size	32
Validation Split	20%
Additional Training Info	Model uses input data for both input and output during training

Comparing with previously suggested autoencoders: The provided autoencoder design by Weberpals et al. (2021) focuses on reconstructing input data through a series of dense encoding and decoding layers, without incorporating additional design features such as dropout, regularization, normalization, and kernel initialization. Similarly, our original autoencoder model also lacks these design features, relying on a straightforward dense layer architecture for encoding and decoding.

D Model Architecture for the Supervised Deep Learning Model

The architecture of supervised model comprised multiple dense layers with 100, 50, 30, and 20 neurons, respectively. These layers utilized ReLU activation and He normal kernel initialization to promote effective learning. To prevent overfitting and enhance the model's generalization capability, we incorporated L2 regularization in the dense layers, specifically in the first layer with a regularization factor of 0.01. This regularization technique penalizes the weights' magnitude, encouraging smaller, more robust weight values.

Furthermore, to mitigate overfitting, dropout layers with a rate of 0.3 were interspersed between the dense layers. Batch normalization was also employed to stabilize the learning process by normalizing the inputs to each layer. The final layer, aimed at binary classification, consisted of a single neuron with sigmoid activation.

The model was compiled with binary crossentropy loss, Adam optimizer, and accuracy as the performance metric. The learning rate was set initially and adjusted during training using a scheduler based on epoch count. Early stopping and checkpointing were implemented to enhance training efficiency and model robustness.

Training was conducted over 150 epochs with a batch size of 32, using a 20% validation split to monitor performance. The model's efficacy was evaluated on the test set, focusing on accuracy and loss metrics. Post-training, we extracted features from the last hidden layer (the second-to-last layer) of the neural network. These features were then incorporated into a propensity score model.

Deep Learning Model Architecture and Hyperparameter Selection: In our study, the architecture of the supervised Deep Learning model was carefully designed based on established best practices and empirical testing. The architecture includes multiple hidden layers with ReLU activation functions, which are known for their efficiency in training deep networks by mitigating the vanishing gradient problem.

To prevent overfitting and enhance model generalization, we incorporated several key components: dropout layers with a dropout rate of 0.3, L2 regularization with a penalty coefficient of 0.01, batch normalization layers to stabilize and accelerate training, and the He normal initializer for kernel initialization, which is particularly suited for layers with ReLU activations. The hyperparameters, such as learning rate and batch size, were fine-tuned using a validation dataset derived from the simulation data.

The number of layers and units per layer were chosen based on preliminary experiments and cross-validation to balance complexity and performance. For instance, the architecture typically included layers with specified units, which provided a good trade-off between model capacity and overfitting risk.

Appendix Table D.1: Model Architecture for the Supervised Deep Learning Model

Layer.Type	Neurons	Activation	Dropout.Rate	Kernel.Initialization
Dense (Input Layer) with L2 (0.01) Regularization	100	ReLU	-	He Normal
Dropout after Input Layer	-	-	0.3	-
Dense	50	ReLU	-	He Normal
Batch Normalization after Dense	-	-	-	-
Dropout after Dense	-	-	0.3	-
Dense	30	ReLU	-	He Normal
Dropout after Dense	-	-	0.3	-
Dense	20	ReLU	-	He Normal
Dropout after Dense	-	-	0.3	-
Dense (Output Layer)	1	Sigmoid	-	Glorot Uniform

Appendix Table D.2: Compilation and Training of the Supervised Deep Learning Model

Property	Value
Loss Function	Binary Crossentropy
Optimizer	Adam
Initial Learning Rate	0.0001
Metrics	Accuracy
Epochs	150
Batch Size	32
Validation Split	20%
Callbacks	Early Stopping, Learning Rate Scheduler, Model Checkpoint, CSV Logger

E Performance Measures in Simulations

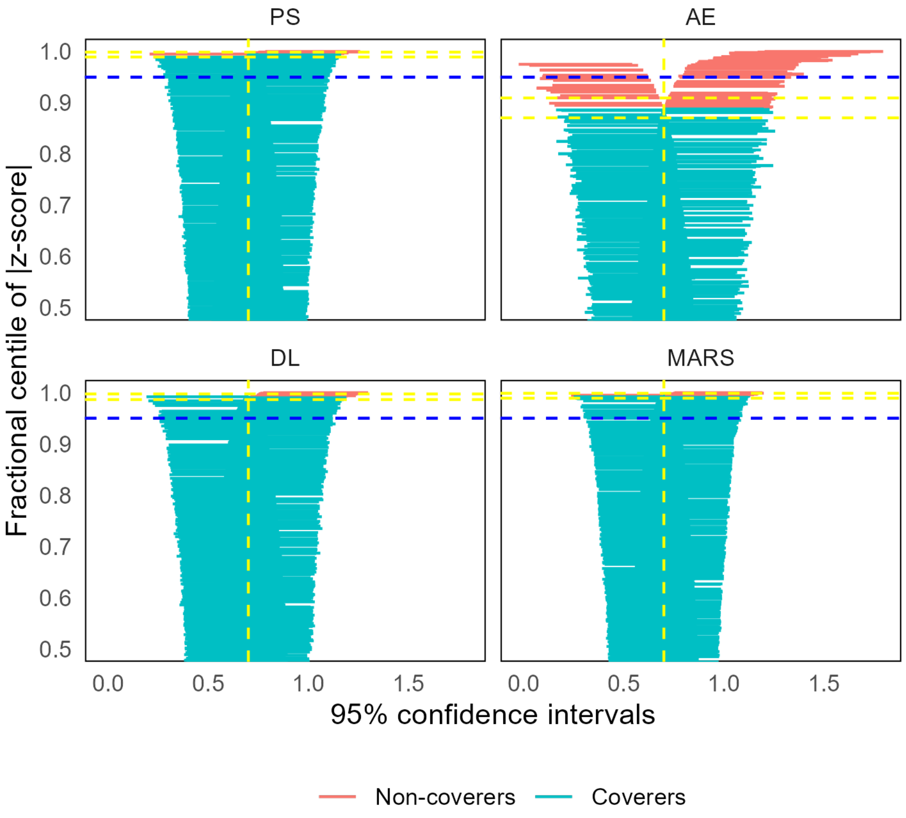

Appendix Figure E.1: Zip plots comparing four propensity score estimation methods in the plasmode simulation in the presence of a frequent exposure (prevalence 30%) and a frequent outcome (prevalence 30%): Logistic Regression (PS), Autoencoders (AE), Deep Learning (DL), and Multivariate Adaptive Regression Splines (MARS). The results are derived from 1,000 sets of plasmode simulation data, each with a sample size of 3,500. The true target parameter was set to an odds ratio of 0.7.

E.1 Frequent Exposure and Frequent Outcome

Our original autoencoder architecture is described in Appendix Table C.1. To enhance the comparability between our deep learning model (‘DL’) and the autoencoder, we introduced several regularization techniques and hyperparameters to the original autoencoder architecture (and named the new model as ‘AE.o’; see Appendix Table E.1). Specifically, we added dropout layers with a dropout rate of 0.3 after each dense layer to prevent overfitting by randomly dropping units during training. We incorporated L2 regularization with a penalty coefficient of 0.01 in the dense layers to penalize large weights and improve generalization. Additionally, we applied batch normalization layers after selected dense layers to normalize the activations and accelerate training convergence. For kernel initialization, we used the He normal initializer for the dense layers to set the initial weights. These modifications ensure that our autoencoder benefits from the same regularization techniques as the deep learning model, providing a robust and fair comparison between the two methodologies.

Our original deep learning architecture is described in Appendix Table D.1. In the analysis, we added a new deep learning model by removing several features to simplify the architecture and ensure a more straightforward comparison with the autoencoder (‘AE’). Specifically, we eliminated dropout layers, which are typically used to prevent overfitting by randomly dropping units during training. We also removed regularization terms, such as L2 regularization, which help to penalize large weights and reduce model complexity. Additionally, we excluded batch normalization layers, which are often used to normalize the input of each layer to improve training stability and speed. Finally, we opted not to use specific kernel initialization methods for the dense layers, which are generally employed to set the initial weights of the model. These changes result in a more basic neural network structure, allowing us to focus on the core performance of the model without the influence of these additional regularization and optimization techniques. We named this model as ‘DL.n’ (see Appendix Table E.1).

Appendix Table E.1: Performance measures of the 6 different propensity score matching methods from the plasmode simulation based on the Right Heart Catheterization (RHC) study in the presence of a frequent exposure (prevalence 30%) and a frequent outcome (prevalence 30%). The results (point Estimate of performance measures, and Monte Carlo Standard Errors) are derived from $1,000$ sets of plasmode simulation data, each with a sample size of $3,500$. The true target parameter was set to an odds ratio of 0.7.

**Performance Measure**	**PS**	**AE**	**AE.o**	**DL**	**DL.n**	**MARS**
Bias	-0.0041 (0.0027)	0.0073 (0.0055) ↑	0.3810 (0.0352) ↑	-0.0023 (0.0029) ↑	0.0749 (0.0034) ↑	-0.0035 (0.0026) ↑
Empirical SE	0.0860 (0.0019)	0.1752 (0.0039) ↑	1.1116 (0.0249) ↑	0.0921 (0.0021) ↑	0.1084 (0.0024) ↑	0.0807 (0.0018) ↓
MSE	0.0074 (0.0003)	0.0307 (0.0018) ↑	1.3797 (0.4361) ↑	0.0085 (0.0004) ↑	0.0174 (0.0009) ↑	0.0065 (0.0003) ↓
Model-based SE	0.1206 (0.0001)	0.1323 (0.0002) ↑	0.2100 (0.0016) ↑	0.1265 (0.0001) ↑	0.1262 (0.0001) ↑	0.1130 (0.0001) ↓
Coverage	0.9940 (0.0024)	0.8900 (0.0099) ↓	0.5830 (0.0156) ↓	0.9920 (0.0028) ↓	0.9410 (0.0075) ↓	0.9940 (0.0024)
Bias-eliminated Coverage	0.9930 (0.0026)	0.8960 (0.0097) ↓	0.3880 (0.0154) ↓	0.9920 (0.0028) ↓	0.9770 (0.0047) ↓	0.9940 (0.0024) ↑

PS = propensity score based on logistic regression; AE = propensity scores based on Autoencoders; DL = propensity scores based on supervised deep learning; AE.o = Optimized version of AE (dropout layers with a rate of 0.3, L2 regularization with a penalty coefficient of 0.01, batch normalization layers, and used the He normal initializer for kernel initialization); DL.n = naive version of DL (simplified by omitting dropout, regularization, normalization, and kernel initialization); MARS = Propensity scores based on Multivariate Adaptive Regression Splines.

SE = Standard Error; MSE = Mean Squared Error; Coverage = Coverage for nominal 95% Confidence Interval.

Arrows indicate the direction of the change in performance measures relative to the PS method: ↑ indicates an increase, and ↓ indicates a decrease.

E.2 Rare Exposure and Frequent Outcome

Appendix Table E.2: Performance measures of the 4 different propensity score matching methods from the plasmode simulation based on the Right Heart Catheterization (RHC) study in the presence of a rare exposure (prevalence 5%) and a frequent outcome (prevalence 30%). The results (point Estimate of performance measures, and Monte Carlo Standard Errors) are derived from $1,000$ sets of plasmode simulation data, each with a sample size of $3,500$. The true target parameter was set to an odds ratio of 0.7.

**Performance Measure**	**PS**	**AE**	**DL**	**MARS**
Bias	-0.0313 (0.0084)	-0.0011 (0.0100) ↑	0.0006 (0.0102) ↑	-0.0325 (0.0080) ↓
Empirical SE	0.2664 (0.0060)	0.3168 (0.0071) ↑	0.3238 (0.0072) ↑	0.2516 (0.0056) ↓
MSE	0.0719 (0.0061)	0.1002 (0.0054) ↑	0.1047 (0.0073) ↑	0.0643 (0.0036) ↓
Model-based SE	0.3400 (0.0009)	0.4000 (0.0010) ↑	0.3918 (0.0010) ↑	0.3263 (0.0007) ↓
Coverage	0.9790 (0.0045)	0.9710 (0.0053) ↓	0.9740 (0.0050) ↓	0.9820 (0.0042) ↑
Bias-eliminated Coverage	0.9780 (0.0046)	0.9710 (0.0053) ↓	0.9740 (0.0050) ↓	0.9780 (0.0046)

PS = propensity score based on logistic regression; AE = propensity scores based on Autoencoders; DL = propensity scores based on supervised deep learning; MARS = Propensity scores based on Multivariate Adaptive Regression Splines.
SE = Standard Error; MSE = Mean Squared Error; Coverage = Coverage for nominal 95% Confidence Interval.
Arrows indicate the direction of the change in performance measures relative to the PS method: ↑ indicates an increase, and ↓ indicates a decrease.

E.3 Frequent Exposure and Rare Outcome

Appendix Table E.3: Performance measures of the 4 different propensity score matching methods from the plasmode simulation based on the Right Heart Catheterization (RHC) study in the presence of a frequent exposure (prevalence 30%) and a rare outcome (prevalence 5%). The results (point Estimate of performance measures, and Monte Carlo Standard Errors) are derived from $1,000$ sets of plasmode simulation data, each with a sample size of $3,500$. The true target parameter was set to an odds ratio of 0.7.

**Performance Measure**	**PS**	**AE**	**DL**	**MARS**
Bias	-0.0078 (0.0061)	-0.0172 (0.0071) ↓	-0.0047 (0.0063) ↑	-0.0079 (0.0055) ↓
Empirical SE	0.1914 (0.0043)	0.2253 (0.0050) ↑	0.2000 (0.0045) ↑	0.1753 (0.0039) ↓
MSE	0.0367 (0.0019)	0.0510 (0.0035) ↑	0.0400 (0.0026) ↑	0.0308 (0.0016) ↓
Model-based SE	0.2605 (0.0006)	0.2733 (0.0007) ↑	0.2660 (0.0005) ↑	0.2396 (0.0004) ↓
Coverage	0.9820 (0.0042)	0.9790 (0.0045) ↓	0.9850 (0.0038) ↑	0.9860 (0.0037) ↑
Bias-eliminated Coverage	0.9810 (0.0043)	0.9780 (0.0046) ↓	0.9840 (0.0040) ↑	0.9860 (0.0037) ↑

PS = propensity score based on logistic regression; AE = propensity scores based on Autoencoders; DL = propensity scores based on supervised deep learning; MARS = Propensity scores based on Multivariate Adaptive Regression Splines.
SE = Standard Error; MSE = Mean Squared Error; Coverage = Coverage for nominal 95% Confidence Interval.
Arrows indicate the direction of the change in performance measures relative to the PS method: ↑ indicates an increase, and ↓ indicates a decrease.

E.4 Large Sample size

Appendix Table E.4: Performance measures of the 4 different propensity score matching methods from the plasmode simulation based on the Right Heart Catheterization (RHC) study in the presence of a frequent exposure (prevalence 30%) and a frequent outcome (prevalence 30%). The results (point Estimate of performance measures, and Monte Carlo Standard Errors) are derived from $1,000$ sets of plasmode simulation data, each with a sample size of $5,000$. The true target parameter was set to an odds ratio of 0.7.

**Performance Measure**	**PS**	**AE**	**DL**	**MARS**
Bias	-0.0047 (0.0023)	0.0050 (0.0052) ↑	-0.0019 (0.0023) ↓	-0.0034 (0.0021) ↓
Empirical SE	0.0712 (0.0016)	0.1630 (0.0036) ↑	0.0743 (0.0017) ↑	0.0663 (0.0015) ↓
MSE	0.0051 (0.0002)	0.0266 (0.0021) ↑	0.0055 (0.0003) ↑	0.0044 (0.0002) ↓
Model-based SE	0.0998 (0.0001)	0.1093 (0.0002) ↑	0.1047 (0.0001) ↑	0.0938 (0.0001) ↓
Coverage	0.9930 (0.0026)	0.8550 (0.0111) ↓	0.9900 (0.0031) ↓	0.9930 (0.0026)
Bias-eliminated Coverage	0.9930 (0.0026)	0.8460 (0.0114) ↓	0.9900 (0.0031) ↓	0.9930 (0.0026)

PS = propensity score based on logistic regression; AE = propensity scores based on Autoencoders; DL = propensity scores based on supervised deep learning; MARS = Propensity scores based on Multivariate Adaptive Regression Splines.

SE = Standard Error; MSE = Mean Squared Error; Coverage = Coverage for nominal 95% Confidence Interval.

Arrows indicate the direction of the change in performance measures relative to the PS method: ↑ indicates an increase, and ↓ indicates a decrease.

E.5 Null Effect

Appendix Table E.5: Performance measures of the 4 different propensity score matching methods from the plasmode simulation based on the Right Heart Catheterization (RHC) study in the presence of a frequent exposure (prevalence 30%) and a frequent outcome (prevalence 30%). The results (point Estimate of performance measures, and Monte Carlo Standard Errors) are derived from $1,000$ sets of plasmode simulation data, each with a sample size of $3,500$. The true target parameter was set to an odds ratio of 1.

**Performance Measure**	**PS**	**AE**	**DL**	**MARS**
Bias	0.0154 (0.0041)	0.0125 (0.0069) ↓	0.0131 (0.0042) ↓	0.0124 (0.0038) ↓
Empirical SE	0.1306 (0.0029)	0.2182 (0.0049) ↑	0.1330 (0.0030) ↑	0.1204 (0.0027) ↓
MSE	0.0173 (0.0009)	0.0477 (0.0030) ↑	0.0178 (0.0009) ↑	0.0146 (0.0007) ↓
Model-based SE	0.1192 (0.0001)	0.1295 (0.0002) ↑	0.1252 (0.0001) ↑	0.1116 (0.0001) ↓
Coverage	0.9320 (0.0080)	0.8000 (0.0126) ↓	0.9320 (0.0080)	0.9390 (0.0076) ↑
Bias-eliminated Coverage	0.9360 (0.0077)	0.7920 (0.0128) ↓	0.9310 (0.0080) ↓	0.9360 (0.0077)

PS = propensity score based on logistic regression; AE = propensity scores based on Autoencoders; DL = propensity scores based on supervised deep learning; MARS = Propensity scores based on Multivariate Adaptive Regression Splines.

SE = Standard Error; MSE = Mean Squared Error; Coverage = Coverage for nominal 95% Confidence Interval.

Arrows indicate the direction of the change in performance measures relative to the PS method: ↑ indicates an increase, and ↓ indicates a decrease.

E.6 Comparing Standard Errors

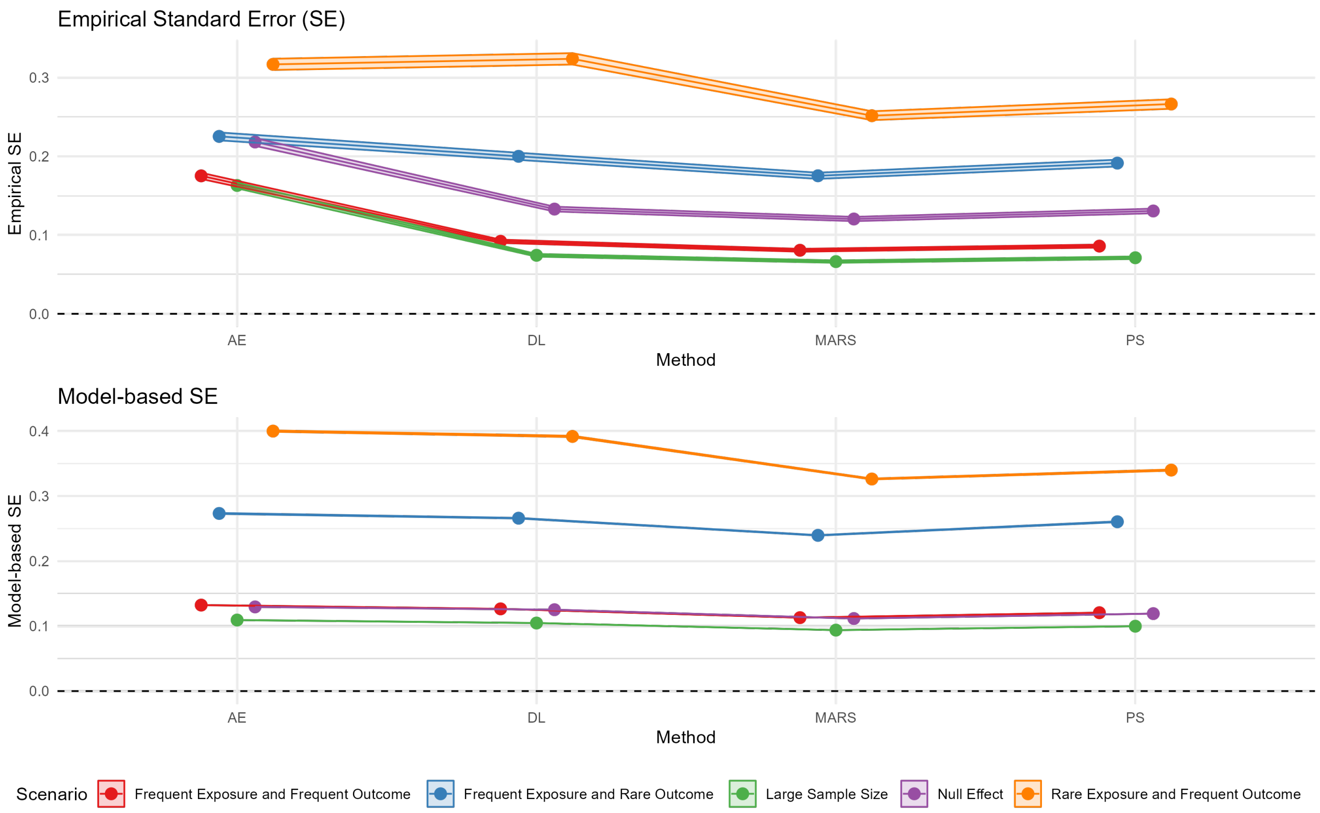

Appendix Figure E.2: Plots comparing empirical and model-based standard errors (point Estimates and Monte Carlo Standard Errors of corresponding performance measures) from four propensity score estimation methods in the plasmode simulation under different scenarios: Logistic Regression (PS), Autoencoders (AE), Deep Learning (DL), and Multivariate Adaptive Regression Splines (MARS). The results are derived from $1,000$ sets of plasmode simulation data.

F Step-by-step Software Guide for Reproducing the Analysis Results

Data Preparation and Summary

Here we show reproducible codes of how one can conduct the analyses described in the manuscript. For the following analysis, we used the following `R` packages: `caret`, `Hmisc`, `factoextra`, `gt`, `gtsummary`, `smd`, `ggplot2`, `dplyr`, `MatchIt`, `cobalt`, `jtools`, `earth`, `keras`, `tensorflow`, `checkmate`, `SuperLearner` and `tmle`.

Data Download

The right heart catheterization (RHC) dataset is obtained from hbiostat.org/data (http://hbiostat.org/data), courtesy of the Vanderbilt University Department of Biostatistics.

```
# Set a seed for reproducibility of random operations
set.seed(123)
# Read the RHC dataset from the URL and store it within R
rhc <- read.csv("https://hbiostat.org/data/repo/rhc.csv")
```

Data Preparation

We converted the treatment variable to a binary indicator to represent the administration of RHC, with ‘RHC’ coded as 1 and all other values as 0. Similarly, the death variable was transformed into a binary outcome variable indicating 30-day mortality, with ‘Yes’ responses coded as 1. The categorical variables (race, sex, primary disease category, cancer status) were then recoded to appropriate categories. Adjustment variables of interest included demographic, clinical, and comorbidity information.

```
# Convert the 'swang1' variable to a binary treatment variable where 'RHC' is encoded as 1 and all other values as 0
rhc$tx  <-  ifelse(rhc$swang1 == "RHC", 1, 0)

# Convert the 'dth30' variable to a binary outcome variable where 'Yes' is encoded as 1 and all other values as 0
rhc$death  <-  ifelse(rhc$dth30 == "Yes", 1, 0)

# Convert the 'race' variable into a factor with specified levels
rhc$race <- factor(rhc$race, levels = c("white", "black", "other"))

# Convert the 'sex' variable into a factor to treat it as a categorical variable
rhc$sex <- as.factor(rhc$sex)
# Relevel the 'sex' factor so that 'Male' is the reference category
rhc$sex <- relevel(rhc$sex, ref = "Male")

# Convert the 'cat1' variable into a factor and merge several categories into 'Other' except for 'ARF', 'CHF', and 'MOSF'
rhc$cat1 <- as.factor(rhc$cat1)
levels(rhc$cat1) <- c("ARF", "CHF", "Other", "Other", "Other", "Other", "Other", "MOSF", "MOSF")

# Convert the 'ca' variable into a factor and redefine its levels
rhc$ca <- as.factor(rhc$ca)
levels(rhc$ca) <- c("Metastatic", "None", "Localized (Yes)")
rhc$ca <- factor(rhc$ca, levels = c("None", "Localized (Yes)", "Metastatic"))

# Create a vector of variable names to be used in the analysis
var.names <- Cs(tx, death, age, sex, race, edu, income, ninsclas, cat1,
                resp, card, neuro, gastr, renal, meta, hema, seps, trauma,
                ortho, das2d3pc, dnr1, ca, surv2md1, aps1, scoma1, wtkilo1,
                temp1, meanbp1, resp1, hrt1, pafi1, paco21, ph1, wblc1,
                hema1, sod1, pot1, crea1, bili1, alb1, cardiohx, chfhx,
                dementhx, psychhx, chrpulhx, renalhx, liverhx, gibledhx,
                malighx, immunhx, transhx, amihx)

# Subset the 'rhc' data frame to include only the variables specified in 'var.names'
rhc2 <- rhc[var.names]
```

Summarizing the data

We prepare the summary table based on the prepared data. The table is stratified by RHC status. We also reported the standardized mean differences for each covariate.

Appendix Table 1: Summary of the variables stratified by the Right Heart Catheterization (RHC) Status, and the associated standardized mean differences.

**Variable**	**Did Not Receive RHC**, N = 3,551[1]	**Received RHC**, N = 2,184[1]	**Difference**[2]	**95% CI**[2,3]
Death	1,088 (31%)	830 (38%)	-0.16	-0.21, -0.10
Age			0.06	0.01, 0.11
Median (IQR)	65 (50, 75)	64 (50, 73)		
Sex			0.09	0.04, 0.15
Male	1,914 (54%)	1,278 (59%)		
Female	1,637 (46%)	906 (41%)		
Race			0.04	-0.02, 0.09
white	2,753 (78%)	1,707 (78%)		
black	585 (16%)	335 (15%)		
other	213 (6.0%)	142 (6.5%)		
Years of Education			-0.09	-0.14, -0.04
Median (IQR)	12.0 (10.0, 13.0)	12.0 (10.0, 14.0)		
Income			0.14	0.09, 0.20
$11-$25k	713 (20%)	452 (21%)		
$25-$50k	500 (14%)	393 (18%)		
> $50k	257 (7.2%)	194 (8.9%)		
Under $11k	2,081 (59%)	1,145 (52%)		
Medical Insurance			0.19	0.14, 0.25
Medicaid	454 (13%)	193 (8.8%)		
Medicare	947 (27%)	511 (23%)		
Medicare & Medicaid	251 (7.1%)	123 (5.6%)		
No insurance	186 (5.2%)	136 (6.2%)		
Private	967 (27%)	731 (33%)		
Private & Medicare	746 (21%)	490 (22%)		
Primary Disease Category			0.55	0.49, 0.60
ARF	1,581 (45%)	909 (42%)		
CHF	247 (7.0%)	209 (9.6%)		
Other	955 (27%)	208 (9.5%)		
MOSF	768 (22%)	858 (39%)		
Respiratory Diagnosis	1,481 (42%)	632 (29%)	0.27	0.22, 0.32
Cardiovascular Diagnosis	1,007 (28%)	924 (42%)	0.29	0.24, 0.35
Neurological Diagnosis	575 (16%)	118 (5.4%)	0.35	0.30, 0.41
Gastrointestinal Diagnosis	522 (15%)	420 (19%)	0.12	0.07, 0.17
Renal Diagnosis	147 (4.1%)	148 (6.8%)	0.12	0.06, 0.17

[1] n (%); RHC: Right heart catheterization

[2] Standardized Mean Difference

[3] CI = Confidence Interval

Appendix Table 1: Summary of the variables stratified by the Right Heart Catheterization (RHC) Status, and the associated standardized mean differences.

**Variable**	**Did Not Receive RHC**, N = 3,551[1]	**Received RHC**, N = 2,184[1]	**Difference**[2]	**95% CI**[2,3]
Metabolic Diagnosis	172 (4.8%)	93 (4.3%)	0.03	-0.03, 0.08
Hematologic Diagnosis	239 (6.7%)	115 (5.3%)	0.06	0.01, 0.12
Sepsis Diagnosis	515 (15%)	516 (24%)	0.23	0.18, 0.29
Trauma Diagnosis	18 (0.5%)	34 (1.6%)	0.10	0.05, 0.16
Orthopedic Diagnosis	3 (<0.1%)	4 (0.2%)	0.03	-0.03, 0.08
Duke Activity Status Index (DASI)			-0.06	-0.12, -0.01
Median (IQR)	19.7 (15.7, 23.5)	19.9 (16.7, 23.4)		
DNR Status on Day 1	499 (14%)	155 (7.1%)	0.23	0.17, 0.28
Cancer Status			0.11	0.05, 0.16
None	2,652 (75%)	1,727 (79%)		
Localized (Yes)	638 (18%)	334 (15%)		
Metastatic	261 (7.4%)	123 (5.6%)		
2-Month Survival Probability			0.20	0.15, 0.25
Median (IQR)	0.64 (0.49, 0.76)	0.60 (0.45, 0.72)		
APACHE Score			-0.50	-0.56, -0.45
Median (IQR)	50 (38, 62)	60 (47, 74)		
Glasgow Coma Score			0.11	0.06, 0.16
Median (IQR)	0 (0, 41)	0 (0, 37)		
Weight (kg)			-0.26	-0.31, -0.20
Median (IQR)	68 (54, 81)	74 (61, 87)		
Temperature (°C)			0.02	-0.03, 0.07
Median (IQR)	38.09 (36.20, 39.00)	38.09 (36.09, 39.00)		
Mean Blood Pressure			0.46	0.40, 0.51
Median (IQR)	68 (53, 119)	57 (47, 73)		
Respiratory Rate			0.17	0.11, 0.22
Median (IQR)	30 (20, 39)	28 (12, 37)		
Heart Rate			-0.15	-0.20, -0.09
Median (IQR)	120 (76, 140)	125 (105, 145)		
PaO2/FiO2 Ratio			0.43	0.38, 0.49
Median (IQR)	224 (149, 333)	168 (110, 267)		
PaCO2			0.25	0.20, 0.30
Median (IQR)	38 (32, 44)	36 (30, 40)		
pH Level			0.12	0.07, 0.17
Median (IQR)	7.40 (7.35, 7.46)	7.40 (7.32, 7.46)		

[1] n (%); RHC: Right heart catheterization

[2] Standardized Mean Difference

[3] CI = Confidence Interval

Appendix Table 1: Summary of the variables stratified by the Right Heart Catheterization (RHC) Status, and the associated standardized mean differences.

Variable	**Did Not Receive RHC**, N = 3,551[1]	**Received RHC**, N = 2,184[1]	**Difference**[2]	**95% CI**[2,3]
White Blood Cell Count			-0.08	-0.14, -0.03
Median (IQR)	14 (8, 19)	15 (9, 21)		
Hematocrit			0.27	0.22, 0.32
Median (IQR)	31 (27, 39)	29 (26, 33)		
Sodium Level			0.09	0.04, 0.15
Median (IQR)	136 (133, 142)	136 (132, 141)		
Potassium Level			0.03	-0.03, 0.08
Median (IQR)	3.80 (3.40, 4.60)	3.80 (3.40, 4.60)		
Creatinine			-0.27	-0.32, -0.22
Median (IQR)	1.30 (0.90, 2.00)	1.80 (1.20, 3.00)		
Bilirubin			-0.14	-0.20, -0.09
Median (IQR)	1.01 (0.70, 1.20)	1.01 (1.00, 1.70)		
Albumin			0.23	0.18, 0.28
Median (IQR)	3.50 (2.70, 3.50)	3.50 (2.40, 3.50)		
History of Cardiovascular Disease	567 (16%)	446 (20%)	-0.12	-0.17, -0.06
Congestive Heart Failure History	596 (17%)	425 (19%)	-0.07	-0.12, -0.02
Dementia History	413 (12%)	151 (6.9%)	0.16	0.11, 0.22
Psychiatric History	286 (8.1%)	100 (4.6%)	0.14	0.09, 0.20
Chronic Pulmonary Disease History	774 (22%)	315 (14%)	0.19	0.14, 0.25
Chronic Renal Disease History	149 (4.2%)	106 (4.9%)	-0.03	-0.08, 0.02
Liver Disease History	265 (7.5%)	136 (6.2%)	0.05	0.00, 0.10
History of GI Bleeding	131 (3.7%)	54 (2.5%)	0.07	0.02, 0.12
Malignancy History	872 (25%)	444 (20%)	0.10	0.05, 0.15
Immunosuppression History	907 (26%)	636 (29%)	-0.08	-0.13, -0.03
History of Hospital Transfer	335 (9.4%)	327 (15%)	-0.17	-0.22, -0.12
History of Acute Myocardial Infarction	105 (3.0%)	95 (4.3%)	-0.07	-0.13, -0.02

[1] n (%); RHC: Right heart catheterization

[2] Standardized Mean Difference

[3] CI = Confidence Interval

Pre-processing the data for deep learning

Dummy variables were generated for categorical features. Continuous variables were preprocessed using centering and scaling to normalize their distributions. The preprocessed data, consisting of binary and normalized continuous variables, was combined into a single dataset.

```
# Create dummy variables for categorical features in 'rhc2' for regression analysis
dmy <- dummyVars(" ~ .", data = rhc2, fullRank = TRUE)

# Apply the dummy variable transformation to 'rhc2' and store the result in a new data frame 'rhc_prep'
rhc_prep <- data.frame(predict(dmy, newdata = rhc2))
```

```
# Exclude the outcome and treatment indicator variables from the feature set
x.var <- names(rhc_prep)[!names(rhc_prep) %in% c("death", "tx")]

# Identify binary variables in the dataset by checking if all their values are either 0 or 1
sel.col <- apply(rhc_prep, 2, function(x) { all(x %in% 0:1) })

# Separate continuous and binary variables based on the above identification
sel.continuous.col <- names(sel.col[sel.col == FALSE])
sel.binary.col <- names(sel.col[sel.col == TRUE])

# Subset the data frame to include only binary variables
rhc_prep1 <- rhc_prep[sel.binary.col]

# Preprocess the continuous variables by centering and scaling them
rhc_prep2fit <- preProcess(rhc_prep[sel.continuous.col], method = c("center", "scale"))

# Apply the preprocessing to the continuous variables
rhc_prep2 <- predict(rhc_prep2fit, rhc_prep[sel.continuous.col])

# Combine the binary and preprocessed continuous variables into a final dataset
rhc_prep3 <- cbind(rhc_prep1, rhc_prep2)
x.var <- setdiff(names(rhc_prep3), c("death", "tx", "id"))

# Add an 'id' column to the final dataset for identification purposes
rhc_prep3$id <- 1:nrow(rhc_prep3)

# Write the final preprocessed dataset to a CSV file for further analysis or sharing
write.csv(rhc_prep3, "rhc3.csv")
```

Analysis

Steps

In all of the following analyses, 4 steps were followed to conduct the propensity core analysis (see Appendix Table 2).

Appendix Table 2: Steps of propensity score analysis.

Step	Description
Step 1	Exposure (RHC) modelling based on identified characteristics/covariates, or some function of them. This modelling can be done using various statistical and machine learning methods to estimate the probability (propensity score) that a patient would receive RHC based on their characteristics. The predicted probabilities from this regressions are the propensity scores.
Step 2	After estimating propensity scores, patients who received RHC are matched with those who did not, based on their propensity scores. We used the nearest neighbor matching method at a 1:1 ratio. A caliper ensures that matched pairs are similar in their probabilities.
Step 3	The balance of covariates in the matched dataset is assessed to ensure that the matching process has successfully created comparable treated and untreated groups. Standardized mean differences (SMD) are commonly used to measure such balance. SMD values below 0.25 was used as an indication of good balance.
Step 4	The final step involves modelling the outcome as a function of the treatment (RHC), while adjusting for covariates. This double adjustment approach, where covariates are adjusted in both the propensity score model and the outcome model, helps to account for any residual confounding.

Details about step 1 (exposure modelling)

We will be using 4 different approaches to estimate the propensity scores in step 1:

1. Logistic regression: A traditional statistical method where the log odds of receiving treatment/being exposed (RHC) are modelled as a linear combination of covariates.
2. Multivariate Adaptive Regression Splines (MARS): A non-parametric regression technique that can model complex, non-linear relationships between the covariates and the treatment assignment.
3. Autoencoders: A type of neural network used for unsupervised learning of efficient codings, which can be used to reduce the dimensionality of covariates and capture non-linear interactions before estimating propensity scores.
4. Deep learning: Utilizing deep neural networks to model the treatment assignment process, capable of capturing complex, non-linear relationships between the covariates and the treatment.

Remaining steps

Rest of the steps (2-4) remain the same after estimating the propensity scores:

Targeted Maximum Likelihood Estimation

We have also added a Targeted Maximum Likelihood Estimation (TMLE) step, so that we can compare our matching estimates with estimates from a double robust approach.

Propensity score matching when propensity scores estimated using a logistic regression

```
# Step 1: Exposure modeling
exposure <- "tx"

# Create a formula for the propensity score model, including all variables in x.var
ps.formula <- as.formula(paste0(exposure, "~", paste(x.var, collapse = "+")))

# Fit a logistic regression model to estimate propensity scores based on exposure and covariates
PS.fit <- glm(ps.formula,
              family = binomial(link = "logit"),  # Use logistic regression
              data = rhc_prep3)

# Predict propensity scores for each observation in the dataset
# Get predicted probabilities (propensity scores)
rhc_prep3$PS <- predict(PS.fit,
                        newdata = rhc_prep3,
                        type = "response")

# Recode exposure variable to a factor with more descriptive labels for plotting
rhc_prep3$RHC <- factor(rhc_prep3$tx, levels = c(0, 1),
                  labels = c("Did Not Receive RHC", "Received RHC"))
```

```
# Create a density plot of propensity scores, colored by RHC status
ggplot(rhc_prep3, aes(x = PS, fill = RHC)) +
        geom_density(alpha = 0.5) +  # Use semi-transparent density plots
        labs(title = "Density Plot of Propensity Scores",
             x = "Propensity Score",
             y = "Density",
             fill = "Exposure Category") +  # Add labels
        theme_minimal()  # Use a minimalistic theme
```

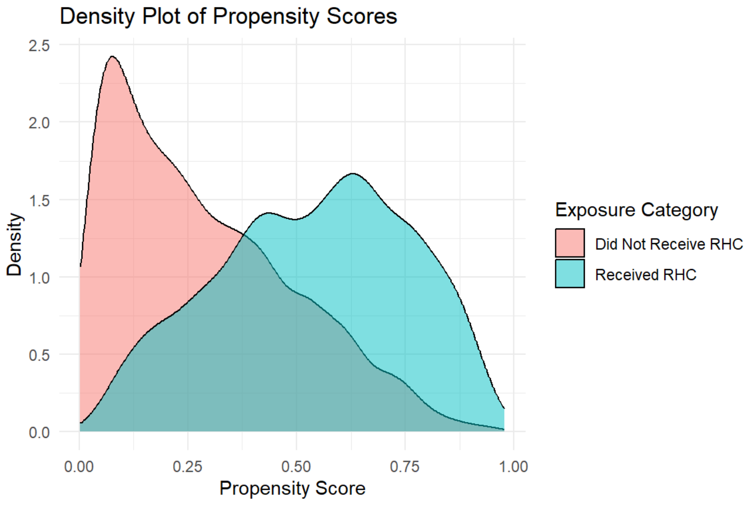


```
# Step 2: Match participants based on propensity scores
logitPS <- -log(1 / rhc_prep3$PS - 1)  # Convert propensity scores to logit scale for matching

# Perform nearest neighbor matching on logit-transformed propensity scores with a caliper
match.obj <- matchit(ps.formula, data = rhc_prep3,
                     distance = 'logit',  # Use logit of PS for matching
                     method = "nearest",  # Nearest neighbor matching
                     replace = FALSE,  # No replacement
                     caliper = .2 * sd(logitPS),  # Set caliper width
                     ratio = 1)  # 1:1 matching

# Store original propensity scores and then remove from data to prevent them from being used as covariates
ps.l <- rhc_prep3$PS
rhc_prep3$PS <- NULL
```

```
# Step 3: Assess balance post-matching to ensure comparability between groups
# Love plot for balance diagnostics
love.plot(match.obj, binary = "std", thresholds = c(m = .25))
```

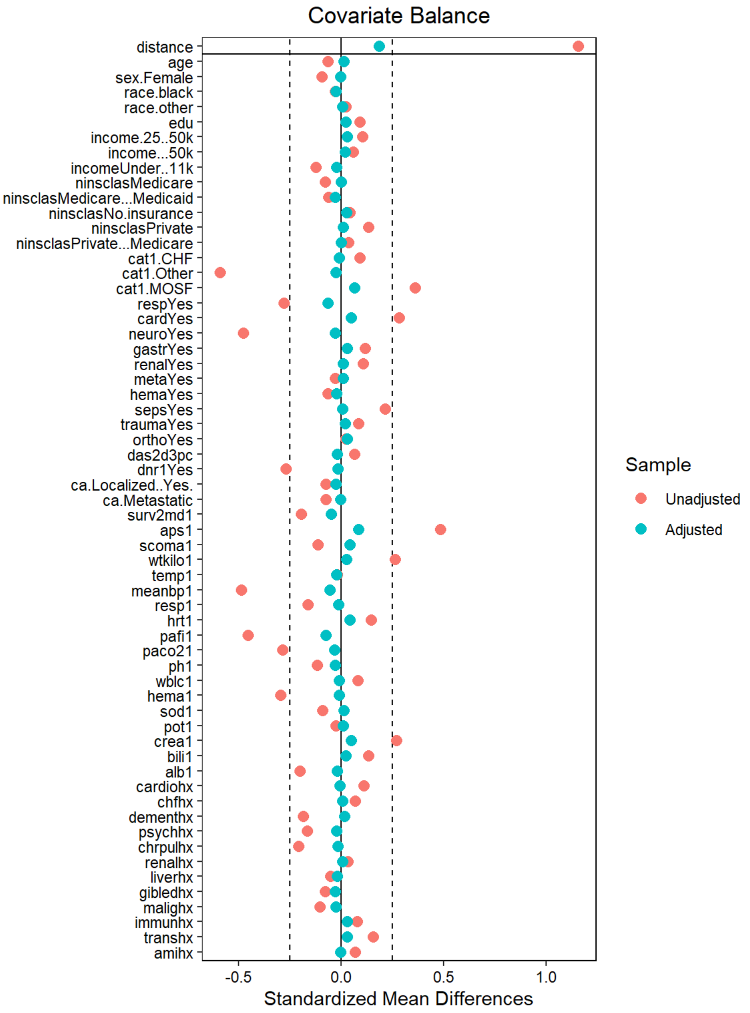


```
# Extract matched dataset for outcome analysis
matched.data.ps <- match.data(match.obj)

# Step 4: Model outcomes using matched data with double adjustment for exposure and covariates
adj <- glm(as.formula(paste0("death", "~", exposure, "+", paste(x.var, collapse = "+"))),
           family = binomial(link = "logit"),  # Logistic regression for binary outcome
           weights = weights,  # Use weights from matching
           data = matched.data.ps)

# Summarize results, using robust standard errors and clustering by match pairs
results.ps <- summ(adj, robust = "HC0", confint = TRUE, digits = 3,
                   cluster = "subclass", model.info = FALSE,
                   model.fit = FALSE, exp = TRUE)$coeftable[exposure,]
```

```
# Targeted Maximum Likelihood Estimation
ObsData.noYA <- rhc_prep3[,x.var]
tmle.fit.ps <- tmle::tmle(Y = rhc_prep3$death,
                       A = rhc_prep3$tx,
                       W = ObsData.noYA,
                       family = "binomial",
                       V.Q = 5,
                       Q.SL.library = c("SL.glm", "SL.earth"),
                       g1W = ps.l,
                       gbound = 5/sqrt(nrow(rhc_prep3))/log(nrow(rhc_prep3)))
estOR.tmle.ps <- tmle.fit.ps$estimates$OR
```

Appendix Table 3: Summary of effect of RHC on death outcome based on two approaches (propensity score matching and Targeted Maximum Likelihood Estimation [TMLE]), when the propensity scores were estimated via a logistic regression. The outcome regression from TMLE was fitted via a super learner (with logistic regression and MARS as candidate learners) with 5 fold cross-validation.

	**Odds Ratio**	**2.5%**	**97.5%**	**SE**	**n**
Propensity score matching	1.40	1.19	1.65	0.08	3,476
TMLE	1.23	1.09	1.40	0.01	5,735

Propensity score matching when propensity scores estimated using Multivariate Adaptive Regression Splines (MARS)

```
# Step 1: Exposure modeling
# Fit a MARS to estimate propensity scores based on exposure and covariates
PS.fit <- earth(ps.formula,
                data=rhc_prep3)

# Predict propensity scores for each observation in the dataset
# Get predicted probabilities (propensity scores)
rhc_prep3$PS <- predict(PS.fit, newdata = rhc_prep3)
ps.e0 <- rhc_prep3$PS
lower_bound <- 0
upper_bound <- 1
rhc_prep3$PS <- pmax(lower_bound, pmin(upper_bound, rhc_prep3$PS))
```

```
# Create a density plot of propensity scores, colored by RHC status
ggplot(rhc_prep3, aes(x = PS, fill = RHC)) +
        geom_density(alpha = 0.5) +  # Use semi-transparent density plots
        labs(title = "Density Plot of Propensity Scores",
             x = "Propensity Score",
             y = "Density",
             fill = "Exposure Category") +  # Add labels
        theme_minimal()  # Use a minimalistic theme
```

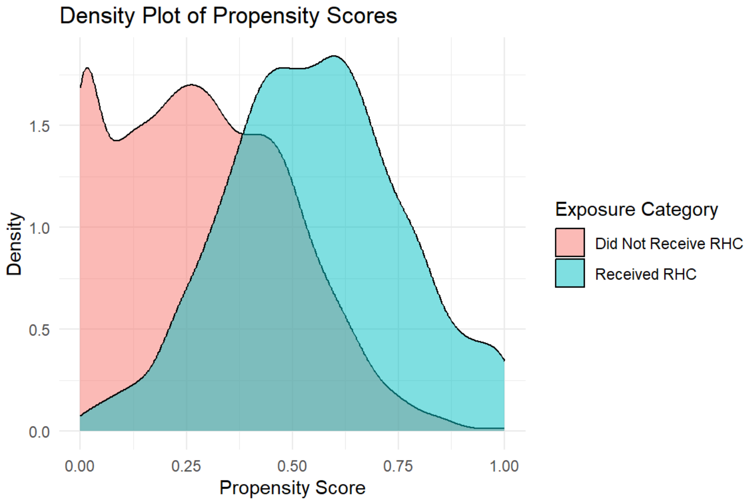


```
# Step 2: Match participants based on propensity scores
logitPS <- -log(1 / rhc_prep3$PS - 1)  # Convert propensity scores to logit scale for matching

# Perform nearest neighbor matching on logit-transformed propensity scores with a caliper
match.obj.e <- matchit(ps.formula, data = rhc_prep3,
                     distance = 'logit',  # Use logit of PS for matching
                     method = "nearest",  # Nearest neighbor matching
                     replace = FALSE,  # No replacement
                     caliper = .2 * sd(logitPS),  # Set caliper width
                     ratio = 1)  # 1:1 matching

# Store original propensity scores and then remove from data to prevent them from being used as covariates
ps.e <- rhc_prep3$PS
rhc_prep3$PS <- NULL
```

```
# Step 3: Assess balance post-matching to ensure comparability between groups
# Love plot for balance diagnostics
love.plot(match.obj.e, binary = "std", thresholds = c(m = .25))
```

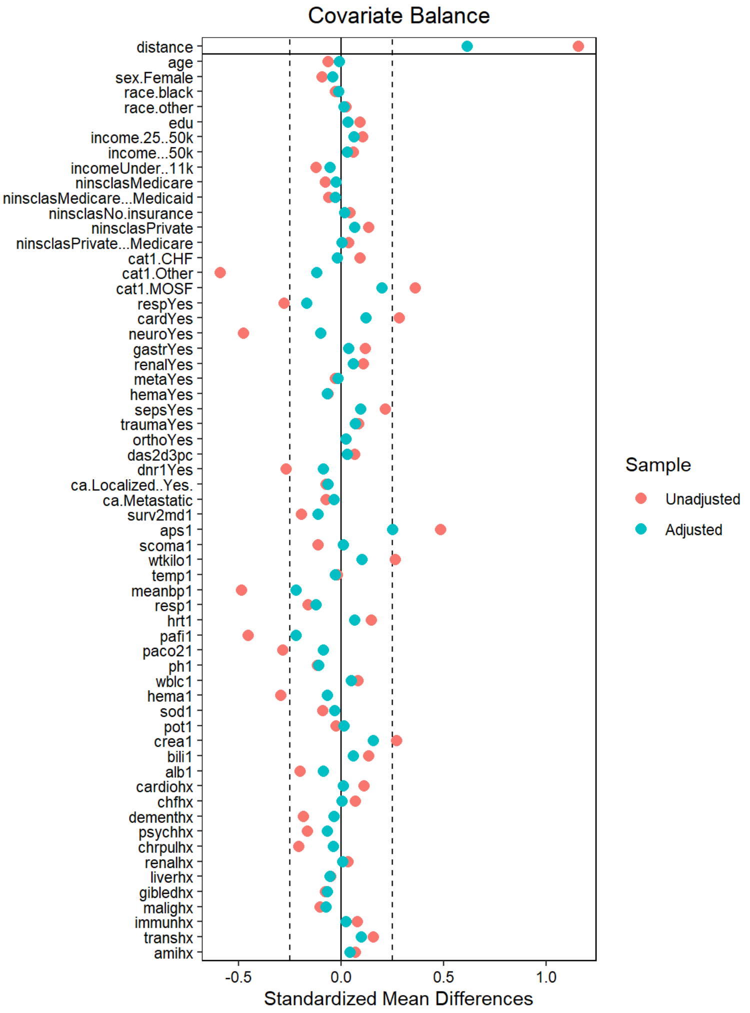


```
# Extract matched dataset for outcome analysis
matched.data.e <- match.data(match.obj.e)

# Step 4: Model outcomes using matched data with double adjustment for exposure and covariates
adj <- glm(as.formula(paste0("death", "~", exposure, "+", paste(x.var, collapse = "+"))),
           family = binomial(link = "logit"),  # Logistic regression for binary outcome
           weights = weights,  # Use weights from matching
           data = matched.data.e)

# Summarize results, using robust standard errors and clustering by match pairs
results.e <- summ(adj, robust = "HC0", confint = TRUE, digits = 3,
                  cluster = "subclass", model.info = FALSE,
                  model.fit = FALSE, exp = TRUE)$coeftable[exposure,]
```

```
# Targeted Maximum Likelihood Estimation
tmle.fit.e <- tmle::tmle(Y = rhc_prep3$death,
                       A = rhc_prep3$tx,
                       W = ObsData.noYA,
                       family = "binomial",
                       V.Q = 5,
                       Q.SL.library = c("SL.glm", "SL.earth"),
                       g1W = ps.e,
                       gbound = 5/sqrt(nrow(rhc_prep3))/log(nrow(rhc_prep3)))
estOR.tmle.e <- tmle.fit.e$estimates$OR
```

Appendix Table 4: Summary of effect of RHC on death outcome based on two approaches (propensity score matching and Targeted Maximum Likelihood Estimation [TMLE]), when the propensity scores were estimated via a Multivariate Adaptive Regression Splines (MARS) regression. The outcome regression from TMLE was fitted via a super learner (with logistic regression and MARS as candidate learners) with 5 fold cross-validation.

	Odds Ratio	2.5%	97.5%	SE	n
Propensity score matching	1.36	1.17	1.57	0.08	4,368
TMLE	1.34	0.98	1.82	0.01	5,735

Propensity score matching when propensity scores estimated using covariates from an Autoencoders

Note: GPU computer needed to conduct the following analysis.

```
initial_lrate <- 0.0001 # 1e-4  # Adjusted Learning rate
K <- backend()
W.data <- rhc_prep3
trainIndex <- createDataPartition(y = W.data[, 1], p = 0.8, list = FALSE)
w_train <- W.data[trainIndex, ]
w_test <- W.data[-trainIndex, ]
w_train <- as.matrix(w_train)
w_test <- as.matrix(w_test)

model <- keras_model_sequential() %>%
  # Encoder Layers
  layer_dense(units = 100, activation = 'relu', input_shape = ncol(w_train)) %>%
  layer_dense(units = 50, activation = 'relu') %>%
  # Bottleneck Layer
  layer_dense(units = 30, activation = 'relu') %>%
  # Decoder Layers
  layer_dense(units = 50, activation = 'relu') %>%
  layer_dense(units = 100, activation = 'relu') %>%
  # Output Layer
  layer_dense(units = ncol(w_train), activation = 'sigmoid')

model %>% compile(
  loss = 'mean_squared_error',
  optimizer = optimizer_adam(learning_rate = initial_lrate)
)

callback_early_stopping <- callback_early_stopping(
  monitor = "val_loss",
  patience = 10,  # Number of epochs with no improvement after which training will be stopped
  restore_best_weights = TRUE  # Whether to restore model weights from the epoch with the best value of the monitored quanti
ty
)

# Learning rate scheduler
decay_rate <- 0.5
decay_steps <- 10000

callback_learning_rate_scheduler <- callback_learning_rate_scheduler(
  function(epoch, lr) {
    if (epoch %% decay_steps == 0 && epoch > 0) {
      lr <- lr * decay_rate
    }
    return(lr)
  }
)
```

```
checkpoint_filename <- paste0("logAE/best_model_", "analyzeAE", ".hdf5")
callback_checkpoint <- callback_model_checkpoint(
  filepath = checkpoint_filename,
  save_best_only = TRUE,
  monitor = "val_loss",
  mode = "min"
)
log_filename <- paste0("logAE/training_log_", "analyzeAE", ".csv")
callback_csv_logger <- callback_csv_logger(log_filename)

historyAE <- model %>% fit(
  x = w_train, y = w_train,  # Using w_train for both input and output
  epochs = 150,
  batch_size = 32,
  validation_split = 0.2,
  verbose = 2,
  callbacks = list(callback_early_stopping,
                   callback_learning_rate_scheduler,
                   callback_checkpoint,
                   callback_csv_logger)
)
```

```
# Evaluate model on test set
metrics <- model %>% evaluate(w_test, w_test)  # Note: Using w_test for both input and output
print(metrics)

current_learning_rate <- K$get_value(model$optimizer$lr)
initial_lrate <- current_learning_rate

# Extract features from the bottleneck layer
bottleneck_layer_model <- keras_model(inputs = model$input, outputs = get_layer(model, index = 6)$output)
#bottleneck_features <- predict(bottleneck_layer_model, w_test)
W.data.matrix <- as.matrix(W.data)
bottleneck_features <- predict(bottleneck_layer_model, W.data.matrix)
bottleneck_features_df <- as.data.frame(bottleneck_features)
colnames(bottleneck_features_df) <- paste0("feature", 1:ncol(bottleneck_features_df))

# Combine with other data
ae.data0 <- cbind(bottleneck_features_df, tx = rhc_prep3$tx, death = rhc_prep3$death)
ae.data <- cbind(ae.data0, rhc_prep3[,x.var])

# Step 1: Exposure modeling
# Create the formula using names from the dataframe obtained from autoencoder results
ae.formula <- as.formula(paste("tx", "~", paste0(names(bottleneck_features_df), collapse = "+")))

ae.fit <- glm(formula = ae.formula, data = ae.data, family = binomial)

# Predict propensity scores for each observation in the dataset
# Get predicted probabilities (propensity scores)
ae.data$PS <- predict(ae.fit, newdata = ae.data, type="response")
```

```
# Create a density plot of propensity scores, colored by RHC status
ggplot(rhc_prep3, aes(x = PS, fill = RHC)) +
        geom_density(alpha = 0.5) +  # Use semi-transparent density plots
        labs(title = "Density Plot of Propensity Scores",
             x = "Propensity Score",
             y = "Density",
             fill = "Exposure Category") +  # Add labels
        theme_minimal()  # Use a minimalistic theme
```

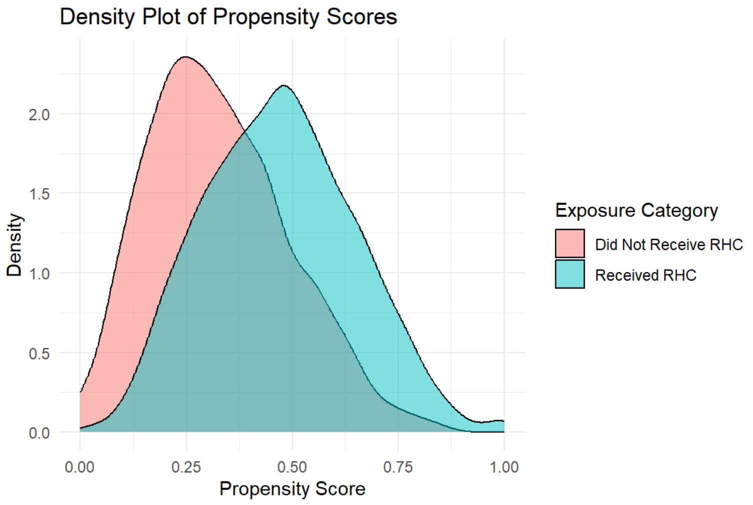


```
# Step 2: Match participants based on propensity scores
logitPS <-  -log(1/ae.data$PS - 1)

# Perform nearest neighbor matching on logit-transformed propensity scores with a caliper
match.obj.ae <- matchit(ae.formula, data = ae.data,
                     distance = 'logit',
                     method = "nearest",
                     replace = FALSE,
                     caliper = .2*sd(logitPS),
                     ratio = 1)

# Store original propensity scores and then remove from data to prevent them from being used as covariates
ps.ae <- ae.data$PS
ae.data$PS <- NULL
```

```
# Step 3: Assess balance post-matching to ensure comparability between groups
# Love plot for balance diagnostics
love.plot(match.obj.ae, binary = "std", thresholds = c(m = .25))
```

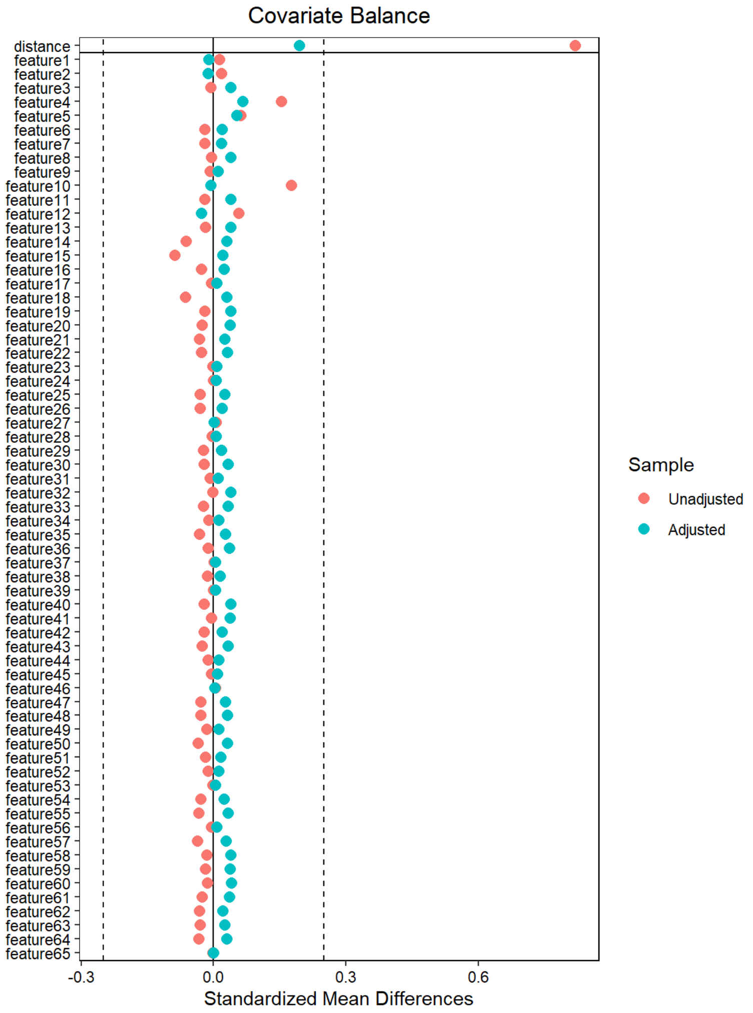


```
# Extract matched dataset for outcome analysis
matched.data.ae <- match.data(match.obj.ae)
p.discard <- (n - nrow(matched.data.ae))/n*100

# Step 4: Model outcomes using matched data with double adjustment for exposure and covariates
adj <- glm(as.formula(paste0("death", "~", exposure, "+", paste(x.var, collapse = "+"))),
           family = binomial(link = "logit"),  # Logistic regression for binary outcome
           weights = weights,  # Use weights from matching
           data = matched.data.ae)

# Summarize results, using robust standard errors and clustering by match pairs
results.ae <- summ(adj, robust = "HC0", confint = TRUE, digits = 3,
                   cluster = "subclass", model.info = FALSE,
                   model.fit = FALSE, exp = TRUE)$coeftable[exposure,]
```

```
# Targeted Maximum Likelihood Estimation
tmle.fit.ae <- tmle::tmle(Y = rhc_prep3$death,
                       A = rhc_prep3$tx,
                       W = ObsData.noYA,
                       family = "binomial",
                       V.Q = 5,
                       Q.SL.library = c("SL.glm", "SL.earth"),
                       g1W = ps.ae,
                       gbound = 5/sqrt(nrow(rhc_prep3))/log(nrow(rhc_prep3)))
estOR.tmle.ae <- tmle.fit.ae$estimates$OR
```

Appendix Table 5: Summary of effect of RHC on death outcome based on two approaches (propensity score matching and Targeted Maximum Likelihood Estimation [TMLE]), when the propensity scores were estimated via trasformed covariates from an autoencoder. The outcome regression from TMLE was fitted via a super learner (with logistic regression and MARS as candidate learners) with 5 fold cross-validation.

	Odds Ratio	2.5%	97.5%	SE	n
Propensity score matching	1.15	0.98	1.36	0.08	4,142
TMLE	1.06	0.92	1.21	0.01	5,735

Propensity score matching when propensity scores estimated using covariates from a deep learner

Note: GPU computer needed to conduct the following analysis.

```
# Setting necessary parameters
initial_lrate <- 0.0001
K <- backend()
W.data <- rhc_prep3
trainIndex <- createDataPartition(W.data$death, p = 0.8, list = FALSE)
y_train <- W.data$death[trainIndex]
y_test <- W.data$death[-trainIndex]
W.data$tx <- NULL
W.data$death <- NULL
W.data$id <- NULL
# W.data$X <- NULL

w_train <- W.data[trainIndex,] # Training data
w_test <- W.data[-trainIndex,] # Testing data
w_train <- as.matrix(w_train)
w_test <- as.matrix(w_test)

model <- keras_model_sequential() %>%
  layer_dense(units = 100, kernel_initializer = 'he_normal',
              activation = 'relu', input_shape = ncol(w_train),
              kernel_regularizer = regularizer_l2(0.01)) %>%
  layer_dropout(rate = 0.3) %>%
  layer_dense(units = 50, kernel_initializer = 'he_normal',
              activation = 'relu') %>%
  layer_batch_normalization() %>%
  layer_dropout(rate = 0.3) %>%
  layer_dense(units = 30, kernel_initializer = 'he_normal',
              activation = 'relu') %>%
  layer_dropout(rate = 0.3) %>%
  layer_dense(units = 20, kernel_initializer = 'he_normal',
              activation = 'relu') %>%
  layer_dropout(rate = 0.3) %>%
  layer_dense(units = 1, kernel_initializer = 'glorot_uniform',
              activation = 'sigmoid') # Output layer for regression

model %>% compile(
  loss = "binary_crossentropy",
  optimizer = optimizer_adam(learning_rate = initial_lrate),
  metrics = c('accuracy')
)

# fit model
callback_early_stopping <- callback_early_stopping(
  monitor = "val_loss",
  patience = 10,
  restore_best_weights = TRUE
)
step_decay <- function(epoch, lr) {
  initial_lrate <- 0.001
  drop <- 0.5
  epochs_drop <- 10.0
  if (epoch %% epochs_drop == 0) {
    lr <- lr * drop
  }
  return(lr)
}
callback_learning_rate_scheduler <- callback_learning_rate_scheduler(step_decay)

checkpoint_filename <- paste0("log/best_model_", "Analysis", ".hdf5")
callback_checkpoint <- callback_model_checkpoint(
  filepath = checkpoint_filename,
  save_best_only = TRUE,
  monitor = "val_loss",
  mode = "min"
)
log_filename <- paste0("log/training_log_", "Analysis", ".csv")

# Create the CSV Logger Callback
callback_csv_logger <- callback_csv_logger(log_filename)
```

```r
# Fit the model with the CSV Logger Callback included
historyDL <- model %>% fit(
  x = w_train,
  y = y_train,
  epochs = 150,
  batch_size = 32,
  validation_split = 0.2,
  verbose = 2,
  callbacks = list(callback_early_stopping,
                   callback_learning_rate_scheduler,
                   callback_checkpoint,
                   callback_csv_logger)
)
```

```r
# Evaluate the performance of the model
metrics <- model %>% evaluate(w_test, y_test)
current_learning_rate <- K$get_value(model$optimizer$lr)

# Extract the output from the last hidden layer
# Get the model's layers
layers <- model$layers

# Filter for dense layers
dense_layers <- Filter(function(layer) grepl("^dense_", layer$name), layers)

# After model training
last_hidden_layer <- get_layer(model, index = length(model$layers) - 1)

# Now create the intermediate layer model
intermediate_layer_model <- keras_model(inputs = model$input, outputs = last_hidden_layer$output)

W.data.matrix <- as.matrix(W.data)
intermediate_output <- predict(intermediate_layer_model, W.data.matrix)
intermediate_output_df <- as.data.frame(intermediate_output)
# Ensure that intermediate_output_df has the correct column names
colnames(intermediate_output_df) <- paste0("feature", 1:ncol(intermediate_output_df))

# Combine with other covariate data
dl.data0 <- cbind(intermediate_output_df, tx = rhc_prep3$tx, death = rhc_prep3$death)
dl.data <- cbind(dl.data0, rhc_prep3[,x.var])

# Step 1: Exposure modeling
# Create the formula using names from the dataframe obtained from deep learning results
dl.formula <- as.formula(paste("tx", "~", paste0(names(intermediate_output_df), collapse = "+")))
dl.fit <- glm(formula = dl.formula, data = dl.data, family = binomial)

# Predict propensity scores for each observation in the dataset
# Get predicted probabilities (propensity scores)
dl.data$PS <- as.numeric(predict(dl.fit, newdata = dl.data, type="response"))
```

```r
# Create a density plot of propensity scores, colored by RHC status
ggplot(rhc_prep3, aes(x = PS, fill = RHC)) +
        geom_density(alpha = 0.5) +  # Use semi-transparent density plots
        labs(title = "Density Plot of Propensity Scores",
             x = "Propensity Score",
             y = "Density",
             fill = "Exposure Category") +  # Add labels
        theme_minimal()  # Use a minimalistic theme
```

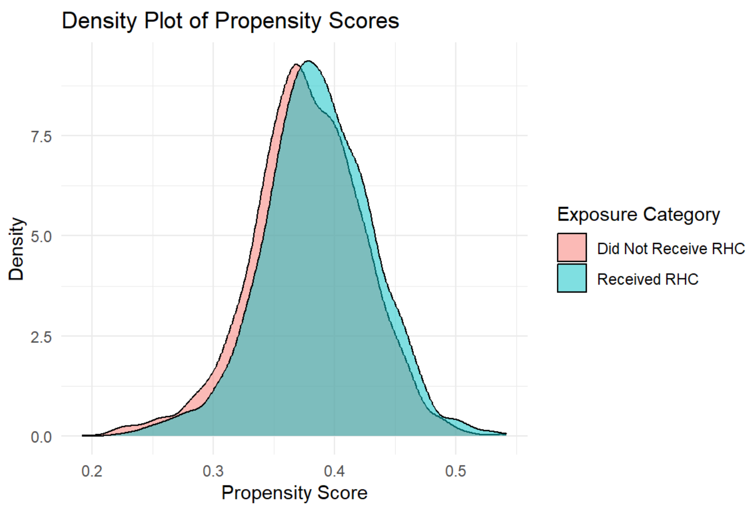


```
# Step 2: Match participants based on propensity scores
logitPS <-  -log(1/dl.data$PS - 1)

# Perform nearest neighbor matching on logit-transformed propensity scores with a caliper
match.obj.dl <- matchit(dl.formula, data = dl.data,
                     distance = 'logit',
                     method = "nearest",
                     replace = FALSE,
                     caliper = .2*sd(logitPS),
                     ratio = 1)

# Store original propensity scores and then remove from data to prevent them from being used as covariates
ps.dl <- dl.data$PS
dl.data$PS <- NULL
```

```
# Step 3: Assess balance post-matching to ensure comparability between groups
# Love plot for balance diagnostics
love.plot(match.obj.dl, binary = "std", thresholds = c(m = .25))
```

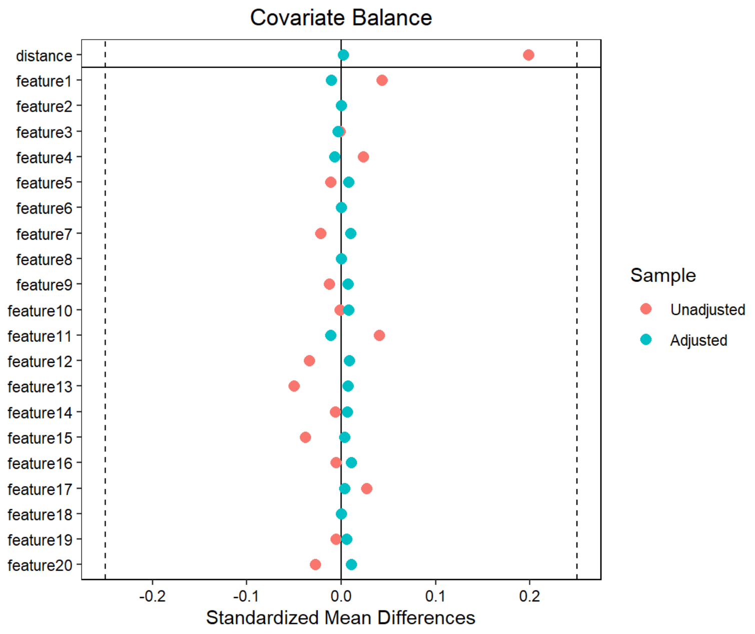


```
# Extract matched dataset for outcome analysis
matched.data.dl <- match.data(match.obj.dl)
p.discard <- (n - nrow(matched.data.dl))/n*100
```

```
# Step 4: Model outcomes using matched data with double adjustment for exposure and covariates
adj <- glm(as.formula(paste0("death", "~", exposure, "+", paste(x.var, collapse = "+"))),
           family = binomial(link = "logit"),  # Logistic regression for binary outcome
           weights = weights,  # Use weights from matching
           data = matched.data.dl)

# Summarize results, using robust standard errors and clustering by match pairs
results.dl <- summ(adj, robust = "HC0", confint = TRUE, digits = 3,
                   cluster = "subclass", model.info = FALSE,
                   model.fit = FALSE, exp = TRUE)$coeftable[exposure,]
```

```
# Targeted Maximum Likelihood Estimation
tmle.fit.dl <- tmle::tmle(Y = rhc_prep3$death,
                          A = rhc_prep3$tx,
                          W = ObsData.noYA,
                          family = "binomial",
                          V.Q = 5,
                          Q.SL.library = c("SL.glm", "SL.earth"),
                          g1W = ps.dl,
                          gbound = 5/sqrt(nrow(rhc_prep3))/log(nrow(rhc_prep3)))
estOR.tmle.dl <- tmle.fit.dl$estimates$OR
```

Appendix Table 6: Summary of effect of RHC on death outcome based on two approaches (propensity score matching and Targeted Maximum Likelihood Estimation [TMLE]), when the propensity scores were estimated via trasformed covariates from an autoencoder. The outcome regression from TMLE was fitted via a super learner (with logistic regression and MARS as candidate learners) with 5 fold cross-validation.

	Odds Ratio	2.5%	97.5%	SE	n
Propensity score matching	1.42	1.20	1.68	0.09	4,334
TMLE	1.24	1.12	1.37	0.01	5,735

Analysis Summary

Odds ratio and associated confidence intervals for RHC from the 4 different approaches of estimating propensity scores. Numerical values are reported in Appendix Tables 3-6.

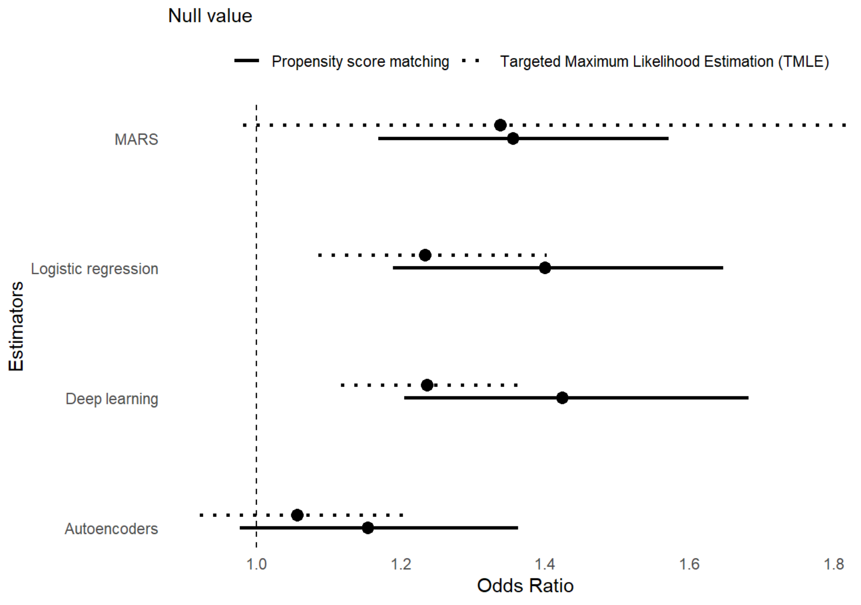

Supplement: Supplementary file 1 — Supplementary Material 1. [file 12874_2024_2284_MOESM1_ESM.pdf]
